# Quantum spins and hybridization in artificially-constructed chains of magnetic adatoms on a superconductor

**DOI:** 10.1038/s41467-022-29879-0

**Published:** 2022-04-20

**Authors:** Eva Liebhaber, Lisa M. Rütten, Gaël Reecht, Jacob F. Steiner, Sebastian Rohlf, Kai Rossnagel, Felix von Oppen, Katharina J. Franke

**Affiliations:** 1 Fachbereich Physik, Freie Universität Berlin, 14195 Berlin, Germany; 2 Dahlem Center for Complex Quantum Systems and Fachbereich Physik, Freie Universität Berlin, 14195 Berlin, Germany; 3 Institut für Experimentelle und Angewandte Physik, Christian-Albrechts-Universität zu Kiel, 24118 Kiel, Germany; 4 Ruprecht Haensel Laboratory, Deutsches Elektronen-Synchrotron DESY, 22607 Hamburg, Germany

**Keywords:** Surfaces, interfaces and thin films, Superconducting properties and materials

## Abstract

Magnetic adatom chains on surfaces constitute fascinating quantum spin systems. Superconducting substrates suppress interactions with bulk electronic excitations but couple the adatom spins to a chain of subgap Yu-Shiba-Rusinov (YSR) quasiparticles. Using a scanning tunneling microscope, we investigate such correlated spin-fermion systems by constructing Fe chains adatom by adatom on superconducting NbSe_2_. The adatoms couple entirely via the substrate, retaining their quantum spin nature. In dimers, we observe that the deepest YSR state undergoes a quantum phase transition due to Ruderman-Kittel-Kasuya-Yosida interactions, a distinct signature of quantum spins. Chains exhibit coherent hybridization and band formation of the YSR excitations, indicating ferromagnetic coupling. Longer chains develop separate domains due to coexisting charge-density-wave order of NbSe_2_. Despite the spin-orbit-coupled substrate, we find no signatures of Majoranas, possibly because quantum spins reduce the parameter range for topological superconductivity. We suggest that adatom chains are versatile systems for investigating correlated-electron physics and its interplay with topological superconductivity.

## Abstract (web summary)

Previous studies of magnetic adatom chains on superconducting substrates have mostly focused on the regime of dense chains and classical spins. Here, using scanning tunnelling microscopy, the authors study the excitation spectra of Fe chains on a NbSe_2_ surface, adatom by adatom, in the regime of quantum spins.

## Introduction

Ever since the early days of quantum mechanics, chains of coupled quantum spins have been a paradigm of strongly interacting quantum systems^1^. Magnetic adatoms on surfaces have been an attractive platform for realizing assemblies of interacting spins, also because atom manipulation allows for designing a wide variety of structures and couplings^2^. On normal-metal substrates, the adatom spins couple to and relax via the particle–hole continuum of the conduction electrons. This relaxation can be suppressed on superconducting substrates as a consequence of their excitation gap^3^. The gap effectively decouples the adatom chain from the bulk electronic excitations of the substrate, while retaining the spin–spin coupling due to the Ruderman–Kittel–Kasuya–Yosida (RKKY)^4–6^ and Dzyaloshinsky–Moriya interactions^7,8^, which are mediated by virtual higher-energy states of the quasiparticle continuum.

Despite the decoupling from bulk excitations of the substrate, chains of magnetic adatoms remain intertwined with localized fermionic subgap excitations. Exchange coupling an adatom spin to the substrate electrons induces discrete Bogoliubov quasiparticles within the superconducting gap known as Yu–Shiba–Rusinov (YSR) excitations^9–12^. Due to the exchange coupling, the adatom binds localized quasiparticles which (partially) screen the adatom spin. Chains of magnetic adatoms on superconductors are thus intriguing many-body systems, which couple quantum spins to an associated chain of well-defined localized fermionic quasiparticles^13^. Such coupled spin–fermion systems are paradigmatic for correlated-electron physics, underlying the physics of Kondo lattice systems^14^ and of high-*T*_c_ superconductors^15^. Unlike these settings, magnetic adatoms on superconductors provide a coupled spin–fermion system which is amenable to bottom-up design in a multitude of geometries.

Chains of magnetic adatoms on superconductors are highly versatile as a result of quantum phase transitions between states with different numbers of bound quasiparticles^16–18^. These transitions arise from the competition between superconducting pairing and the exchange coupling between adatom spin and substrate^16,19–22^, and are associated with discrete changes in the effective adatom spin due to Kondo-like screening. In chains of magnetic adatoms, different screening states have different spin–spin interaction energies, leading to rich magnetic phase diagrams as a function of the binding energy of screening YSR quasiparticles and spin–spin interactions^13^. The competition between screening and spin–spin interactions is specific to quantum spins and absent in models based on classical spins. Intriguingly, the magnetic phase diagrams can be probed directly by tunneling experiments at subgap bias voltages. Tunneling locally binds or unbinds a quasiparticle, introducing a sudden change of the local effective adatom spin. The response to this local perturbation provides signatures in the tunneling spectra which are characteristic of the magnetic ordering^13^.

Here, we report experiments on magnetic chains built Fe adatom by Fe adatom on a 2*H*-NbSe_2_ substrate, a layered superconductor. Due to the quasi-two-dimensional structure, the YSR resonances have a larger spatial extent^23,24^, which facilitates coupling along the chain even for well-separated Fe adatoms. We track the spatially resolved excitation spectra all the way from the monomer to longer chains. Quantum phase transitions induced by the RKKY coupling reveal the intimate coupling of the quantum spins of the adatoms to the fermionic subgap quasiparticles. The YSR excitations hybridize coherently along the chain and the resulting formation of band-like subgap excitations with increasing chain length suggests ferromagnetic coupling between adatom spins. Longer chains are affected by the charge-density-wave (CDW) order of the 2*H*-NbSe_2_ substrate that coexists with superconductivity.

Previous studies of magnetic chains on superconducting substrates were motivated by the search for topological superconductivity and Majorana bound states. These experiments relied on densely packed adatoms, formed either by self assembly^25–29^ or by atom manipulation^30–34^. The theoretical description of these chains assumes classical spin structures and bands of hybridizing adatom *d* orbitals, which cross the Fermi level^25,35–37^. Importantly, the chains investigated here are in a very different regime, previously studied only for YSR dimers^11,38–44^. We construct magnetic adatom chains in the dilute limit^45^, in which the adatoms are spaced sufficiently far that direct hybridization of their *d* orbitals is negligible. The adatoms couple only via spin–spin interactions mediated by the substrate as well as hybridization of their YSR excitations. In this limit, subgap bands emerge from correlated spin–fermion dynamics and are in general not of single-particle nature.

Dilute chains of magnetic adatoms have also been predicted to host topological superconductivity and Majorana end states^13,45–48^. These studies assumed classical spin textures, neglecting the quantum nature of the adatom spin. Quantum spins, however, can have dramatically reduced parameter space exhibiting topological superconductivity^13^, and we do not observe signatures of Majorana end states in any of our chains. In general, one can realize a wide variety of quantum spin chains by tuning the effective adatom spins, the magnetic anisotropy, or the sign and magnitude of the RKKY coupling. While higher-spin adatoms promise more extensive topological superconducting phases, lower-spin adatoms promise more pronounced signatures of correlated electron physics. This makes dilute adatom chains exciting model systems for correlated electron physics, for topological superconductivity, and for their interplay.

## Results

**Fig. 1:**
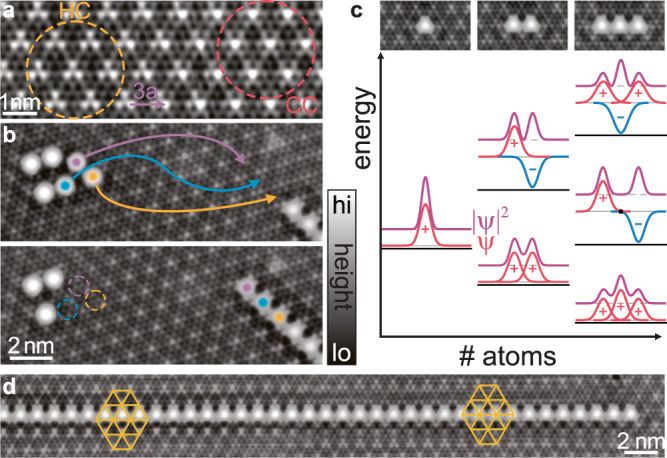
Assembly of Fe chains on NbSe_2_. **a** Atomic-resolution topography of a clean NbSe_2_ surface. Regions with HC (CC) CDW patterns are indicated in yellow (red). **b** Topography images illustrating the sequence of atomic-manipulation steps to obtain an Fe chain. **c** STM images of Fe monomer, dimer, and trimer along with a schematic illustration of YSR hybridization. YSR wave functions of the monomers (taken as Gaussian shaped) with red and blue indicating positive and negative amplitude in overall hybridized wave function ${\psi}$ (with ${\left|{{\psi}} \right|}^{2}$ shown in purple). A tight-binding-like hybridization into linear combinations of monomer wave functions predicts characteristic maxima and nodal planes of the YSR wave function. **d** Topography of a long chain. The yellow grids illustrate the position of the CDW maxima relative to the atoms of the chain. Constant-current set point for all topographies 100 pA, 10 mV.

**Fig. 2:**
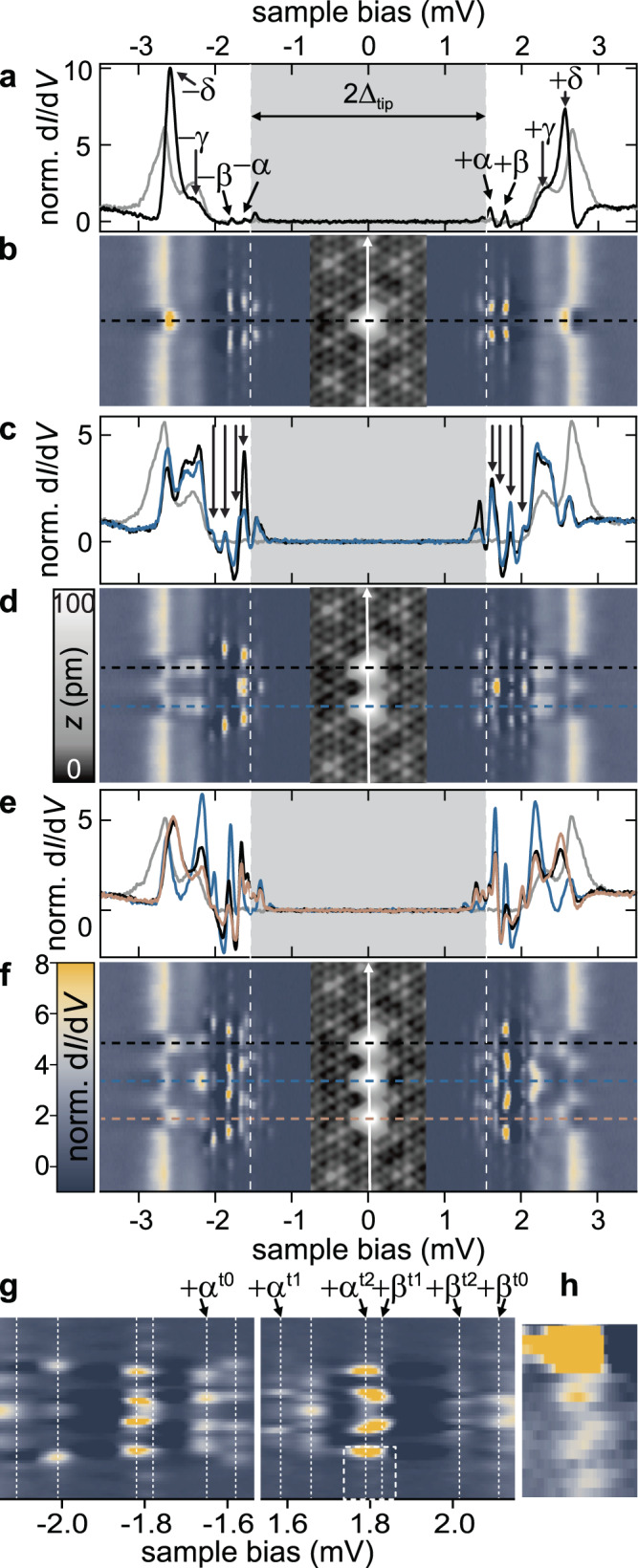
Evolution of d*I*/d*V* spectra from monomer to trimer. **a**, **c**, **e** Normalized differential conductance spectra recorded on the atoms for the monomer **a**, dimer **c**, and trimer **e** and on the bare NbSe_2_ (gray). Tip gap indicated in gray. Feedback was opened at 250 pA, 5 mV and a modulation of 15 μV was used. In **a** the *α*-, *β*-, *γ*- and *δ*-resonances are indicated by black arrows. In **c**, the split resonances from *α* and *β* are indicated by arrows. **b**, **d**, **f** Line profiles recorded along the monomer **b**, dimer **d**, and trimer **f** as illustrated in the topographies (insets; recorded in constant-current mode with set point 100 pA, 10 mV). Horizontal dashed lines serve as guide to the eye for the precise location of the spectra of **a**, **c**, **e**. Vertical white dashed lines indicate 2Δ_tip_. **g** Zoom to subgap range of **f**. The *α*- and *β*-resonances are indicated by the white dashed lines and labeled above the panels. **h** Close-up of the oscillatory decay around 1.8 mV as indicated by the box in **g**.

We build chains on the van der Waals-layered superconductor 2*H*-NbSe_2_ starting with a single Fe atom and continuing all the way to a 51-atom chain. The NbSe_2_ substrate forms a CDW^49–51^ which is reflected in periodic height modulations superimposed on the atomic corrugation (see STM image in Fig. 1a). The CDW is weakly incommensurate with a period of ~3*a* (here, *a* is the lattice constant of NbSe_2_), so that the CDW shifts slowly relative to the atomic lattice. When the CDW maximum is centered on the hollow site (hollow centered, HC), the topography exhibits a three-petaled shape (yellow area in Fig. 1a), while the Se-atom centered configuration (chalcogen centered, CC) exhibits a petalless pattern (red area). Superconductivity appears below a critical temperature of 7.2 K. The anisotropic superconducting gap of about 1 meV results in two-humped coherence peaks in the d*I*/d*V* spectra (see gray spectrum in Fig. 2a)^52–55^.

As deposited, Fe atoms adsorb in the two distinct hollow sites of the terminating Se layer, with or without Nb atom underneath (metal centered and hollow centered, respectively). The two kinds of Fe adatoms exhibit distinct YSR spectra, reflecting different crystal-field splittings as well as exchange and potential scatterings^56^. To maximize YSR hybridization, we assemble shorter chains such that all Fe atoms are positioned on identical (hollow-centered) sites (Fig. 1b, c).

For individual Fe atoms, local variations associated with the CDW shift the energy and modify the wave functions of the YSR states^56^. Consequently, effective hybridization of YSR states requires not only that the adatoms are located at identical adsorption sites of the atomic lattice, but also at comparable positions with respect to the CDW. For this reason, we place hollow-centered Fe adatoms on a sequence of maxima of the CDW, resulting in a dilute chain with an adatom distance of 3*a*. We show that with this choice, the YSR excitations of adjacent Fe atoms are sufficiently similar that they hybridize into symmetric and antisymmetric linear combinations (see sketch in Fig. 1c). Successively placing Fe atoms at a distance of 3*a* (Fig. 1b), we find that chains with up to about ten adatoms remain essentially uniform. Longer chains become inhomogeneous due to the incommensurate nature of the CDW (Fig. 1d).

### Monomer

We begin with an analysis of the monomer. YSR states have been observed in NbSe_2_ for magnetic atoms located in the bulk^23,57^ and on the surface^24,56,58^. A differential conductance (d*I*/d*V*) spectrum of a Fe monomer at the specified adsorption site and recorded with a superconducting Nb tip is shown in Fig. 2a (black line; tip gap indicated in gray). We resolve four pairs of subgap YSR resonances labeled *α*−*δ* at symmetric bias voltages. These emerge from exchange coupling four half-filled *d* orbitals to corresponding conduction electron channels^18,59^, indicating that the Fe atom is in a spin-2 state.

Due to the Kondo-like screening, the effective spin *S*_eff_ of the monomer depends on the number *Q* of bound quasiparticles. Depending on the strength of the exchange coupling within the channel, each channel can bind a quasiparticle or not, with the two ground states separated by a quantum phase transition (see also Supplementary Note 1). Each bound quasiparticle partially screens the bare impurity spin *S* = 2. We can deduce the screening state of the four channels by tracking how the energies of the YSR states shift as a function of the adsorption site relative to the CDW^56^. Our analysis indicates that for Fe on the maximum of the CDW, three YSR resonances (*α*−*γ*) are in the screened state. For the fourth resonance (*δ*), the assignment is less definitive, with the unscreened state being more likely and assumed in the following. With three channels binding a quasiparticle at the impurity site, the spin of the monomer is effectively reduced to ${S}_{{{{{{{{\rm{eff}}}}}}}}}=\frac{1}{2}$, and the monomer is in a $(Q=3,{S}_{{{{{{{{\rm{eff}}}}}}}}}=\frac{1}{2})$ ground state. (Indications against the less likely assignment of four screened channels are provided in the Supplementary Note 2).

The small effective spin ${S}_{{{{{{{{\rm{eff}}}}}}}}}=\frac{1}{2}$ of the adatoms indicates that the spin needs to be treated quantum mechanically. Direct evidence for the quantum spin nature is provided by a Kondo resonance which the Fe atoms induce, when the substrate is in the normal state (see Supplementary Note 4 and Supplementary Fig. 4). We will see below that a description in terms of quantum spins is also strongly suggested by the spectra of adatom dimers on the superconducting substrate.

**Fig. 3:**
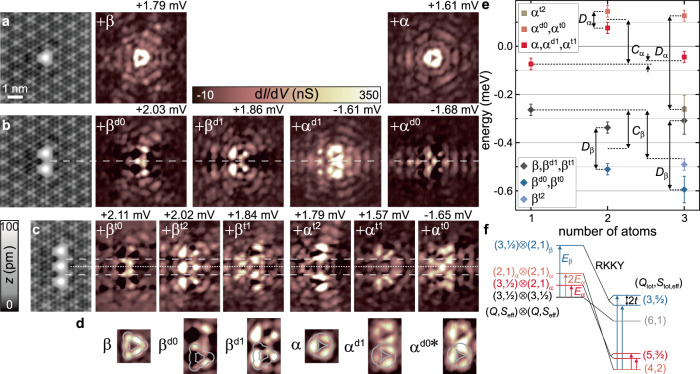
YSR wave functions of monomer, dimer and trimer. **a–c** STM topographies (constant-current mode with set point 100 pA, 10 mV) of one to three Fe atoms with spacing of 3*a* in the left. Corresponding constant-contour d*I*/d*V* maps of the (hybridized) YSR states in the monomer (top row), dimer (middle row) and trimer (bottom row). Constant-contour feedback was opened at 250 pA, 5 mV and a modulation of 15 μV was used. Horizontal dashed/dotted lines serve as guide to the eye. **d** Close-ups around the atoms' center for the monomer and dimer *α* and *β* states. Gray lines serve as guide to the eye. **e** Quantitative energy evolution of the *α* and *β* states upon hybridization. Shift *C* relative to the monomer and splits *D* are indicated. Note that the CDW-induced energetic evolution of the monomer YSR states suggests *α* and *β* to be in the screened-spin regime^56^, hence, implying a negative ground state energy (of the monomer states). The position of the *α* and *β* states were determined from deconvolved spectra, which were fit by the appropriate number of Gaussian peaks. The error bars were determined from the standard deviation of the fit, the error margin of the energy gap of the tip, the modulation voltage of the lock-in, and the sampling interval of the data used for the deconvolution, for details see Supplementary Note 6. **f** Schematic energy level diagram of the dimer. States of the uncoupled dimer (left) are labeled by (*Q*, *S*_eff_) for both adatoms. States of the coupled dimer (right) are labeled by the total number of bound quasiparticles and effective spin, (*Q*_tot_, *S*_tot,eff_). The values of *S*_tot,eff_ assume ferromagnetic RKKY coupling as suggested by band formation in chains (see text).

We can obtain fingerprints of the YSR resonances through their wave-function patterns, which will allow us to track how YSR resonances shift and hybridize as adatoms are assembled into chains. As a result of the quantum phase transitions, one must take some care when interpreting YSR patterns. In the unscreened state (no bound quasiparticle), the electron-like wave function is observed at positive bias voltage and the hole-like wave function at negative biases. In contrast, the assignments are reversed in the screened state. Theoretically, the screened (*E*_YSR_ < 0) and unscreened (*E*_YSR_ > 0) states are characterized by different signs of the YSR energies, and we use this representation when interpreting our data, see, e.g., Fig. 3e.

Throughout this paper, we plot the hole-like YSR wave function. (The maps of the electron-like components are shown in the Supplementary Figs. 7–12.) Specifically, we focus on the YSR states *α* and *β*, which lie deepest inside the gap and are most easily disentangled from the background. Corresponding spectra taken across the Fe atom along the high-symmetry direction of the substrate display long-range oscillations of the YSR wave functions (color plot in Fig. 2b), which have their highest intensity slightly away from the center of the Fe atom. Differential conductance maps of the *α* and *β* resonances exhibit overall *D*_3_ symmetry of the YSR wave functions (brownish plots in Fig. 3a) reflecting the anisotropic Fermi surface of NbSe_2_, the local crystal field, and the symmetry of the CDW^56^.

### Dimer

We realize dimers by placing a second hollow-centered Fe atom an adjacent maximum of the CDW at a distance of 3*a*. Taking d*I*/d*V* spectra at the centers of the two atoms (black and blue lines in Fig. 2c), we now find two states each in the energy regions of the original *α* and *β* states. The doubling of the YSR states persists all along the dimer axis with oscillatory intensity distribution and overall mirror symmetry about the center of the dimer (Fig. 2d). This doubling can be naturally understood in terms of hybrid YSR excitations, which are symmetric and antisymmetric linear combinations of the monomer excitations.

We corroborate this interpretation by d*I*/d*V* maps of the YSR wave functions taken at the corresponding peak energies in Fig. 3b. Two maps (2.03 and 1.86 mV) can be assigned to the hybridized *β* states. This assignment is supported by the shapes of the YSR wave functions, see close-ups in Fig. 3d. The *β* state exhibits intensity at the sides and vertices of a triangular shape. Both features reappear in the patterns of the dimer, albeit with characteristic modifications due to hybridization. The map at 2.03 mV shows increased intensity in between the Fe atoms and is identified with the symmetric combination. The map at 1.86 mV exhibits a nodal line of suppressed intensity perpendicular to the dimer axis (dashed line in Fig. 3b), consistent with the antisymmetric combination. We label these dimer (*d*) states as ${\beta }^{{{{dn}}}}$, where *n* counts the number of nodal planes.

We also identify symmetric and antisymmetric combinations of the *α* states, which appear as a peak with a shoulder in spectra taken directly above the Fe atoms (Fig. 2c) and can be disentangled as two distinct resonances by the corresponding modulations along the dimer axis (Fig. 2d). For this assignment of the symmetric and antisymmetric combination, we inspect the corresponding d*I*/d*V* maps at positive and negative bias voltage (shown in Fig. 3b and Supplementary Fig. 7, respectively). d*I*/d*V* maps of *α*^d0^ and *α*^d1^ indeed lack or exhibit a nodal line perpendicular to the dimer axis.

Surprisingly, we observe that the characteristic spatial shapes have switched bias polarity. Inspection of the d*I*/d*V* pattern probed at negative bias in the close vicinity of the Fe atoms reveals shapes which are strikingly similar to the monomer’s *α* state at positive bias voltage. This is seen most clearly from the close-ups of *α* as well as *α*^d0*^ and *α*^d1^ shown in Fig. 3d. (Note that for *α*^d0^, we present the d*I*/d*V* map recorded at the energy of the corresponding thermal peak as indicated by the asterisk in *α*^d0*^, because the map at *α*^d0^ is strongly influenced by the negative differential conductance of *α*^d1^, see Supplementary Fig. 7). This reflects that the *α* state has crossed the quantum phase transition upon dimer formation. In the monomer, the *α* state is screened and its hole-like wave function is observed at positive bias. In the dimer, the *α*-derived hole-like wave functions appear at negative bias voltage and are therefore unscreened. The quantum phase transition in the *α* states from screened to unscreened is associated with an increase of the effective spin of each Fe atom from ${S}_{{{{{{{{\rm{eff}}}}}}}}}=\frac{1}{2}$ to *S*_eff_ = 1.

The crossing of the quantum phase transition between monomer and dimer requires a substantial shift of the *α* state, in addition to the hybridization splitting. Moreover, this shift is opposite to that of the *β* state, which moves toward the gap edge in the dimer (see Fig. 3e for a quantitative analysis of the shift (*C*) and splitting (*D*) based on deconvolved d*I*/d*V* spectra shown in Supplementary Fig. 5). Previous works^39,42^ have analyzed these shifts and splittings in terms of a classical-spin model. However, neither shifts larger than the splitting nor shifts in opposite directions for different YSR states appear naturally in this model. While both shift and splitting emerge from the hybridization between the YSR excitations of the two adatoms, the splitting appears in first order in the coupling, while the shift appears only in second order^11^ (see theoretical considerations in Supplementary Note 1 for further discussion).

In contrast, a large shift can appear naturally for quantum spins. In this case, unbinding the quasiparticle bound in the *α* channel costs YSR energy, but also increases the effective spin of the Fe adatom from ${S}_{{{{{{{{\rm{eff}}}}}}}}}=\frac{1}{2}$ to *S*_eff_ = 1. This increase in the adatom spins implies that the dimer gains a larger RKKY energy. On balance, it can thus be energetically favorable for the dimer to unbind the quasiparticles, as we observe for the *α*-derived states.

This is further illustrated in the schematic level diagram shown in Fig. 3f, which emphasizes the effects of RKKY coupling and hybridization by showing the states of the uncoupled dimer on the left and of the coupled dimer on the right. In the ground state of the uncoupled dimer, both adatoms are in the $(Q=3,{S}_{{{{{{{{\rm{eff}}}}}}}}}=\frac{1}{2})$ state. Exciting the *α* or *β* resonance of one of the adatoms excites it into (*Q* = 2, *S*_eff_ = 1) states. When only one of the two adatoms is excited, there are two degenerate states of the uncoupled dimer, which are split in the coupled dimer by the YSR hybridization. Such a splitting does not exist for states in which both adatoms are in the same state, resulting in a nondegenerate configuration. In addition to the splittings, there is a downward shift of the energies of the coupled dimer due to the RKKY interaction. This shift is larger for larger *S*_eff_. Thus, as illustrated in Fig. 3f, the ground state of the coupled dimer can emerge from an excited state of the uncoupled dimer, consistent with our observation of a quantum phase transition of the *α* resonance from the monomer to the dimer.

The essential difference between models of classical and quantum spins is that in quantum models, the quantum phase transitions are associated with Kondo-like screening of the adatom spin. Thus, binding a quasiparticle changes the spin state of the adatom only in a quantum model (see also Supplementary Note 1). For instance, when screening a spin-$\frac{1}{2}$ impurity, it forms a singlet with the quasiparticle spin and no longer points in a preferred direction. This contrasts with classical-spin models, in which the impurity spin remains aligned along a certain direction, merely binding a quasiparticle with antiparallel spin.

### Trimer

Forming a trimer by deliberately adding a third atom at a distance of 3*a*, we now identify three states each in the *α* and *β* regions (see spectra in Fig. 2e, f). We can again disentangle the underlying hybrid states based on energy considerations, the intensity modulations of the split states along the trimer axis (see close-ups in Fig. 2g, h), and their corresponding d*I*/d*V* maps (Fig. 3c; in addition to the number *n* of nodal planes, we label the trimer by *t*). Despite the complexity of the patterns due to the oscillatory nature of the YSR wave functions, we tentatively identify the number of nodal planes perpendicular to the trimer axis in the individual maps of the YSR resonances. In the maps at 1.57 and 1.84 mV, we find a single nodal plane at the center of the trimer marked by a dotted line. Two nodal planes between the Fe atoms, marked by dashed lines, are clearly seen in the map at 2.02 mV and less pronounced at 1.79 mV (see also maps at negative bias in Supplementary Fig. 7). The latter state is very close in energy to the state at 1.84 mV and may thus be partially obscured. The maps at 2.11 and −1.65 mV lack clear evidence of nodal planes. We can now assign resonances to *α* and *β* (see Fig. 3c) based on their energy, supported by a comparison of structural elements of their maps to monomer and dimer. In particular, the near field of *β*^t2^ closely resembles the ones of *β* and *β*^d1^. We note that we assign the binding energy of the resonance *α*^t0^ to have opposite sign relative to the others. This assignment is corroborated by the observation that during band formation with increasing chain length, *α*-derived states appear near zero energy (see below).

Our tentative assignments allow us to track the energy shifts and splittings from deconvolved data, which we find to be consistent with the scenario of quantum spins subject to RKKY interactions and hybridization (Fig. 3e). While the RKKY interaction leads to further shifts, hybridization increases the number of states. Importantly, the energy of the hybrid *β* states is not monotonous with the number of nodal planes as we find the *β*^t2^ state in the energetic center of the respective triplet structure. This change in energetic order can be rationalized by accounting for strong next-nearest-neighbor RKKY interactions which arise naturally in a quantum spin model.

### Evolution of YSR bands in Fe atom chains

**Fig. 4:**
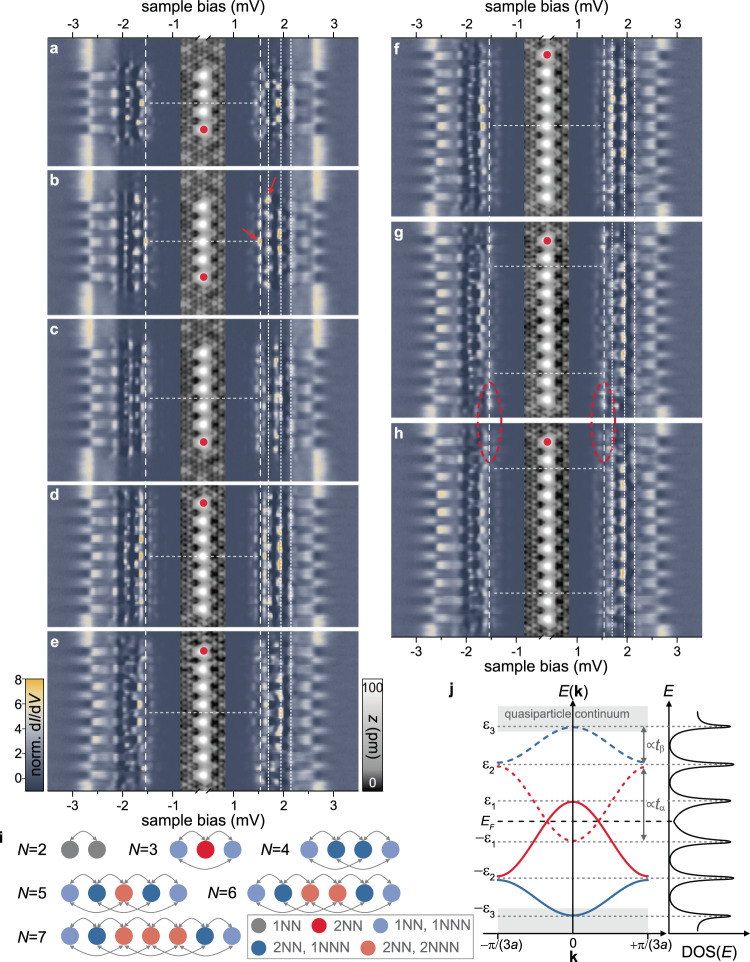
Evolution of YSR bands in Fe atom chains. **a–h** Stacked constant-height d*I*/d*V* spectra (normalized) recorded along lines across chains containing 4–11 atoms as illustrated in the inset topographies (constant-current mode with set point 100 pA, 10 mV). Set point for spectra 250–700 pA, 5 mV with a modulation of 15 μV. Vertical dashed and dotted lines indicate Δ_tip_ ≈ ±1.55 meV and 2.15, 1.95, and 1.7 mV corresponding to energies of 0.6, 0.4, and 0.15 meV, respectively. These energies mark the van Hove singularities of the *α* and *β* derived bands (only at positive bias for clarity), see text. The bands originating from the *γ* and *δ* states are located within the coherence peaks and contribute to the modulations along the chain at higher biases. Horizontal dashed lines in **a**–**f** indicate mirror symmetry axes perpendicular to the chain. Horizontal dashed lines in **g**, **h** delimit the homogeneously appearing bulk of the chain. The states at the chain’s termination are marked by a red ellipse. Red dots mark the atom, that is added to the chain. Red arrows in **b** mark the large intensity at the center of the chain (left) and at the chain’s termination (right), see text. **i** Schematic illustration of the number of (next) nearest (N)NN neighbors for different atoms in chains with different lengths. **j** Schematic illustration of band formation and DOS with van Hove singularities at band edges. ±*ε*_1,2,3_ correspond to the three pairs of dotted lines in **a**–**h**. The solid (dashed) lines represent the dispersion of the electron-like (hole-like) bands of the *α* (red) and *β* (blue) bands.

Figure 4a–h shows chains made up of *N* = 4–11 Fe atoms (the atom added last is marked in red) and corresponding d*I*/d*V* spectra along their entire lengths. Neighboring atoms are placed on hollow sites with a distance of 3*a*, and all adsorption sites remain close to the CDW maximum. We note that the adatoms may help to lock the CDW to the underlying atomic lattice over longer distances than for the pristine surface (cf. Fig. 1a, d).

In the following, we will discuss the modifications of the subgap spectra when increasing the length of the chain by the addition of individual Fe atoms. While our energy resolution no longer allows us to resolve all hybrid states derived from the individual *α*−*δ* resonances, we find that the addition of single adatoms leads to changes of the states along the entire chain. Additional evidence for the delocalized nature of the subgap excitations can be obtained from the preservation of symmetries about the center of the chain as well as the convergence of spectral weight to certain energies with increasing chain length.

For the shorter chains, we observe strong modifications of the subgap spectra with the addition of every atom. These modifications become less pronounced with increasing chain length. In the presence of next-nearest-neighbor interactions, the two outermost atoms at either end of the chain are subject to different interactions from the central atoms (Fig. 4i). Thus, beginning only with *N* = 5 atoms, the chains develop a uniform central region which eventually leads to the formation of band-like YSR excitations. (We do not preclude even longer-range interactions, but do not identify corresponding signatures within our resolution.)

Consistent with this picture, the subgap structure of the quadrumer (Fig. 4a) differs drastically from that of the trimer (Fig. 2f). The *α* and *β* states of the monomer are now expected to form four hybrid states each. However, a detailed assignment becomes difficult as the states begin to overlap. Attaching a fifth atom causes yet another strong modification of the subgap structure (Fig. 4b). For instance, unlike for *N* = 4, we now observe a delocalized state of *α* character at zero energy (i.e., at a bias of ~±1.55 mV corresponding to the tip gap). Apart from the atomic corrugation, this state has a nodeless intensity distribution exhibiting highest spectral weight at the center of the chain and falling off toward both ends (left red arrow, Fig. 4b, d*I*/d*V* maps are provided in Supplementary Fig. 8). Furthermore, there are several resonances around ±1.7 mV which are absent for *N* = 4 such as the prominent state with strong intensity at the chain ends (right red arrow).

Further collective changes appear upon addition of another Fe atom (*N* = 6). Now, the zero-energy state is shifted to slightly higher energies. At ~± 1.7 mV there is a very weak resonance with different spatial fingerprints (no intensity at the chain terminations) as opposed to *N* = 5 (Fig. 4c). The concerted variation of the subgap structure along the entire chain indicates coherent coupling of all YSR excitations. This persists for the longer chains. Within the uniform center of the chain (horizontal dashed lines in panels g, h), the principal features converge into specific energy intervals as indicated by vertical dotted lines (Fig. 4). This behavior is consistent with the formation of band-like YSR excitations originating from the original *α* and *β* excitations of the monomer. Once the band-like behavior is fully developed, the associated van Hove singularities become the strongest features in the d*I*/d*V* spectra. The interpretation of coherent band formation is further supported by the overall mirror symmetry about the center of the chain (cf. dashed lines in Fig. 4a–f).

The van Hove singularities allow one to extract information on the bandwidths. Each resonance of the monomer contributes an electron-like and a hole-like band of the chain. We suggest that the *β*-derived bands extend from the edge of the superconducting gap approximately halfway to the center (solid and dashed blue lines in the sketch of Fig. 4j for electron-like and hole-like bands, respectively). This assignment is based on following the uppermost and lowermost hybrid states of *β*-character in the d*I*/d*V* maps (see Supplementary Note 8 and Supplementary Figs. 8 and 9). We find that the electron-like band appears at negative energies, as the *β*-derived states do not undergo a phase transition with increasing chain length.

Determining the width of the *α*-derived band requires tracking of its parent states for the entire sequence of chain lengths. For intermediate chain lengths, we observe *α*-derived states close to zero energy (e.g. for *N* = 5, see Fig. 4b). This implies that the *α*-derived band crosses zero energy, so that the upper van Hove singularity of the electron-like band appears at ~1.7 mV, i.e., at positive energies. The identification of the lower band edge is less unequivocal. We observe only three van Hove singularities (at fixed bias polarity), while four would be generically expected for two bands. Thus, the *α*-derived band may either lie symmetrically around the Fermi level with band edges at ~±1.7 mV (in which case, its two van Hove singularities would be observed at the same bias voltage), or reach down to the upper van Hove singularity of the *β*-derived band (in which case, the second van Hove singularity of the *α* band would coincide with a van Hove singularity of the *β* band). The latter scenario (red line in Fig. 4j) is more natural in view of the large observed splitting of the *α* states in the trimer (cf. Fig. 3e; note that even in the trimer, the lowest-energy *β* state is already close to the highest-energy *α* state). (To demonstrate the *α*-like character of the bands, we present several d*I*/d*V* maps in Supplementary Figs. 8 and 9.)

It is interesting to note that as a result of the reduced hybridization, the bandwidths of the YSR bands in our dilute chains are considerably smaller than in densely spaced chains^35^, confining bands to lie within the superconducting gap. This precludes the observation of individual states as recently done for dense chains of Mn adatoms on Nb(110), giving patterns reminiscent of particle-in-a-box states^60^.

Crossing of YSR bands through the Fermi level—as we observe for the *α*-derived band—s a necessary requirement for realizing topological superconductivity and Majorana end states^35,45^. Here, we neither find signatures of Majoranas nor of the required opening of a *p*-wave gap around the Fermi energy. The observation of a zero-energy state localized at the chain ends for *N* = 10 (Fig. 4g, red ellipses) disappears upon addition of another Fe atom (Fig. 4h) and should thus be attributed to small spectral changes at the terminations of finite chains (see Supplementary Fig. 9 for d*I*/d*V* maps).

We emphasize that the formation of bands is not a priori obvious for chains of quantum spins. Coupling of the quantum spins along with the associated subgap YSR quasiparticles in general constitutes a many-body problem^13^. Exciting a YSR resonance by tunneling into the quantum spin chain locally modifies the effective spin^13^. For instance, exciting the (screened) *β* resonance increases the effective spin of the local adatom by 1/2, introducing a mobile defect into the spin chain. For a ferromagnetic spin background, the mobile defect effectively leads to a band-like spectral function with van Hove singularities. As illustrated by model calculations for a spin-$\frac{1}{2}$ chain shown in Supplementary Note 2, other spin orders typically exhibit more involved excitation spectra. Our observation of YSR bands and van Hove singularities therefore suggests that the adatom spins couple ferromagnetically. (We note that strictly speaking, we cannot distinguish ferromagnetic coupling from weak spiral modulations as discussed for instance in ref. ^61^.) We also note that consistent with the data, our model calculations reveal strong spectral variations as a function of *N* for short chains which eventually evolve into band-like behavior in longer chains.

### Longer Fe chains and CDW

**Fig. 5:**
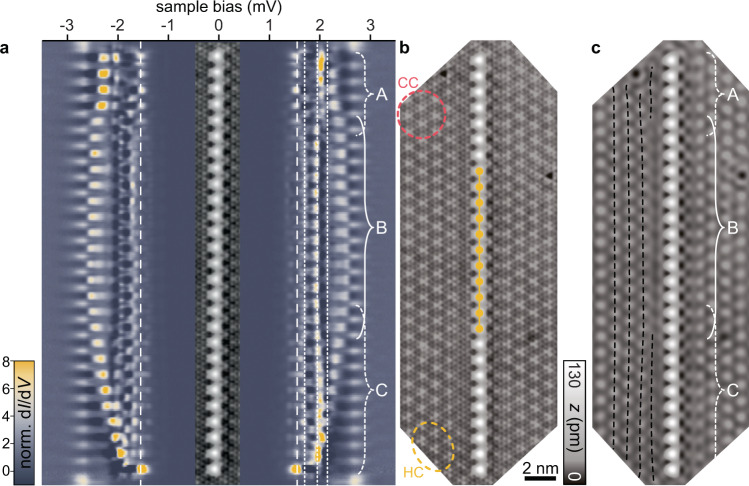
Band banding in longer Fe chains. **a** Stacked constant-height d*I*/d*V* spectra (normalized) recorded along a line across the 27-atom chain as illustrated in the inset topography. Set point for the spectra is 700 pA, 5 mV with a modulation of 15 *μ*V. The (Nb) tip gap Δ_tip_ ≈ 1.55 meV is indicated by white dashed lines. White dotted lines are at the same energies as in Fig. 4 and indicate the van Hove singularities of the *α*- and *β*-derived bands. **b** Larger range STM topography of the 27-atom chain. The position of the former 11-atom chain (Fig. 4h) is indicated in yellow (constant-current set point is 100 pA, 10 mV). A hollow-centered (HC) region of the CDW is highlighted by a yellow circle, a Se-centered (CC) region is marked in red. **c** A FFT-filter has been applied to the STM topography in **b** to remove the atomic corrugation. Dashed lines on the left of the chain serve as guide to the eye to illustrate the CDW order in the vicinity of the chain. Different sections of the chain are labeled with A–C.

For longer chains beyond *N* = 11, the incommensurate nature of the CDW becomes increasingly relevant and the atoms can no longer all sit on maxima of the CDW. d*I*/d*V* spectra recorded along a 27-atom chain (Fig. 5a) reveal spatial variations of the YSR band structure. In the center of the chain (region B), the spectra are similar to those of the 11-atom chain (original 11-atom chain is indicated by yellow circles). However, the YSR bands that fall within the coherence peaks in region B (indicated in Fig. 5a) shift continuously toward the Fermi level at both ends of the chain (more pronounced in region C than in region A; for an assignment of the character of the bands, see Supplementary Figs. 10 and 11). The effect of the CDW is also reflected in the loss of overall mirror symmetry about the center of the chain, which was present in shorter chains (cf. horizontal dashed lines in Fig. 4a–f).

The bending of the YSR bands can be correlated with changes of the CDW along the chain (Fig. 5b, c). The CDW appears as apparent height modulations in the STM topography, which are superimposed on the atomic corrugation (see Fig. 5b). To highlight the CDW, we remove the atomic lattice from the STM image by Fourier filtering (Fig. 5c) and connect maxima of the CDW by black dashed lines (shown only to the left of the chain for clarity). In region A, the CDW is distorted by the dark defect visible at the top. In region B, the CDW runs parallel to the chain and appears locked to the atomic lattice, with all Fe atoms located on CDW maxima. In region C, the CDW does not run parallel to the chain (see additional dashed line), resulting in variations in the adsorption sites of the Fe atoms with respect to the CDW. As shown previously^56^, such changes in the adsorption site shift the YSR states of individual Fe atoms due to variations in the density of states and the potential scattering. Correspondingly, the CDW imposes a smoothly varying potential onto the YSR bands, which causes the YSR bands to bend. Interestingly, the YSR bands close to the superconducting gap edge appear to be more strongly affected. The bands eventually shift to zero energy at one end of the chain (region C). The apparent near-zero-energy state (see Supplementary Figs. 10 and 11) should not be interpreted as a Majorana mode and arises from the effective band bending.

**Fig. 6:**
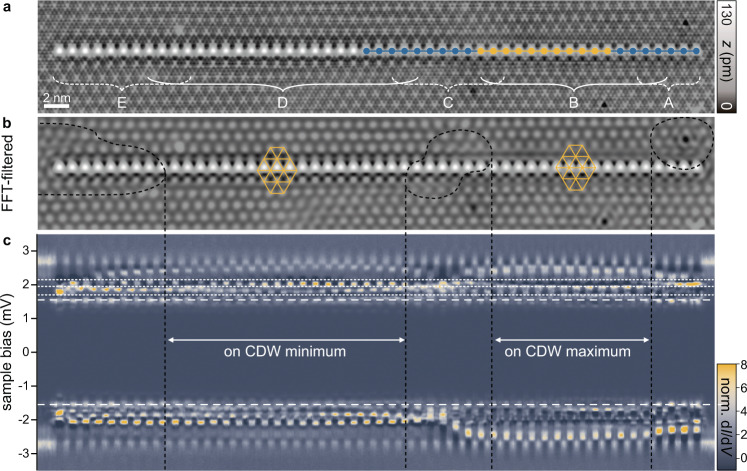
YSR bands across different domains of the CDW. **a** STM topography (constant-current mode with set point 100 pA, 10 mV) of the 51-atom chain. The position of the former 11 (27)-atom chain (Figs. 4h and 5) is indicated in yellow (blue). Different sections of the chain are labeled with A–E. Sections A and B are identical to Fig. 5. **b** To remove the atomic corrugation of the STM image **a** was FFT-filtered. Dashed lines encircle the most pronounced CDW distortions in the background. The yellow grids indicate the CDW and the positions of atoms within the chain relative to the CDW: crossings of the lines lie on CDW maxima, upwards pointing triangles on CDW minima. **c** Stacked constant-height d*I*/d*V* spectra (normalized) recorded along a line across the 51-atom chain (set point 700 pA, 5 mV with a modulation of 15 μV). The tip gap Δ_tip_ ≈ 1.55 meV is indicated by dashed lines. The dotted lines are located at the same energies as in Fig. 4.

The robustness of the band bending can be probed by further increasing the chain length. Figure 6 shows a 51-atom chain, which was created by adding Fe atoms to the 27-atom chain in regions D-E. While the d*I*/d*V* spectra along the chain remain unchanged in regions A and B, the CDW in region C changes during manipulation (see also d*I*/d*V* maps in Supplementary Fig. 12). Moreover, the d*I*/d*V* spectra in regions D and E differ significantly from those in region B. Here, the Fe atoms are located on CDW minima as indicated by yellow triangles in the Fourier-filtered topography in Fig. 6b, where the YSR spectrum of the monomers is characteristically different. Slow variations of the bands along the chain can again be attributed to changes in the relative alignment with respect to the CDW, with the Fourier-filtered image revealing local distortions of the CDW pattern (most pronounced distortions encircled by black dashed lines).

## Discussion

Dilute chains of magnetic adatoms on superconductors are qualitatively distinct from dense adatom chains which were previously investigated in searches for topological superconductivity and Majorana bound states and in which bands of adatom *d* orbitals are believed to cross the Fermi energy of the substrate superconductor. In dilute chains, the quantum spins associated with magnetic adatoms induce subgap quasiparticle excitations, and the substrate-induced coupling between adatoms involves spin–spin coupling via the RKKY and Dzyaloshinsky–Moriya interactions as well as hybridization of subgap quasiparticle excitations. As a result, dilute chains provide an intriguing model system probing the correlated dynamics of the quantum spins of the adatoms and the fermionic subgap quasiparticles.

We have realized such chains using atom manipulation with the STM tip, placing Fe atoms such that they reside on maxima of the CDW, and have probed the spatially resolved excitation spectra all along these chains. By carefully analyzing the subgap spectra exploiting both the YSR energies and YSR wave functions, we uncover evidence for the interactions between quantum spins and fermionic subgap excitations. In particular, we find that RKKY interactions are instrumental in inducing a quantum phase transition. The underlying competition between the energy of the YSR excitation and the RKKY coupling between adatom spins exists only for quantum spins and is absent for classical-spin models. Moreover, our observation of hybridization extending throughout longer chains and the ensuing formation of band-like subgap excitations suggests that the coupling between adatom spins is ferromagnetic. Our results therefore confirm that dilute chains of magnetic adatoms are not only interesting as potential platforms for the observation of Majorana bound states, but also as highly flexible realizations of a broad class of correlated spin–fermion systems. The adatom spacing as well as the orientation relative to the crystallographic axes constitute interesting parameters to be explored. Clearly, this is not restricted to chains, but could be readily extended to two-dimensional arrangements. In both cases, it might be particularly interesting to realize antiferromagnetic RKKY coupling by judiciously adjusting the inter-adatom distance.

The incommensurate nature of the CDW in NbSe_2_ does not play a significant role up to intermediate chain lengths (≲10 nm) as the Fe atoms help to lock the phase of the CDW into registry with the chain. It is only for yet longer chains that the energy stored in these distortions of the CDW can no longer be sustained, triggering an abrupt change of the CDW into a different phase. Adatom chains extending across CDW domain walls exhibit significantly different band structures. In regions of smooth changes of the CDW, the YSR bands shift continuously in energy. More generally, one can exploit substrates structured by charge density waves or by moiré lattices to investigate domain walls between different parameter regimes of the adatom chain. Another intriguing substrate property may be a pair-density wave as putatively observed for NbSe_2_^62^. This might, however, require a different substrate as its effect is presumably negligible for NbSe_2_, where it modulates the superconducting gap with the same wave vector as the CDW and by <1%^62^.

It would clearly be highly desirable to complement our experiments by spin-resolved STM measurements. Normal-metal-based magnetic tips have much lower resolution, making it difficult to resolve the spin polarization of the subgap resonances. A promising path forward would exploit a superconducting tip with a magnetic impurity, with its magnetization direction stabilized by a sufficiently strong magnetic field. Such a magnetic tip would be suitable for high-resolution measurements of YSR states^60^. However, care has to be taken that the magnetic field does not affect the superconductivity of the substrate, e.g., by inducing vortices or reducing the gap, thereby modifying the delicate balance between magnetic and superconducting energies of the dilute chains.

## Methods

Bulk 2*H*-NbSe_2_ crystals were grown by iodine vapor transport^52^ and cleaved under ultra-high vacuum conditions. Fe atoms were deposited on the freshly cleaved sample at temperatures below 15 K. Scanning tunneling microscopy and spectroscopy experiments were performed at <1.2 K. We used superconducting Nb tips with a superconducting energy gap of Δ_tip_ ≈ 1.55 meV. These were prepared by indenting a NbTi wire into a Nb(111) substrate. The superconducting tip effectively increases the energy resolution beyond the temperature-limited Fermi–Dirac distribution. The convolution of tip and sample density of states leads to a shift of all spectral features by the tip’s energy gap. To determine the precise position of the subgap states, we deconvolve the spectra and fit them by the appropriate number of Gaussian peaks, for details see Supplementary Note 6. Importantly, Nb tips are suitable for controlled atom manipulation, which allows for atom-by-atom construction of the Fe chains on the NbSe_2_ substrate and for tracing the spectral evolution upon chain extension.

Note added: After submission of our manuscript another paper investigating adatom chains in the dilute limit has been posted^63^.

## Supplementary information

Supplementary Information

## Data Availability

All data needed to evaluate the conclusions in the paper are present in the paper and the Supplementary Information. The STM data generated in this study have been deposited in the Refubium database under accession code 10.17169/refubium-34026.

## Code availability

The code for the theoretical calculations shown in the Supplementary Information has been deposited in the Refubium database under accession code 10.17169/refubium-34026.
