# Supplementary material for: Quantum spins and hybridization in artificially-constructed chains of magnetic adatoms on a superconductor
Source: Nat Commun. 2022 Apr 20;13:2160. doi: 10.1038/s41467-022-29879-0 (PMC9021194; doi:10.1038/s41467-022-29879-0)
Supplement: Supplementary file 1 — Supplementary Information [file 41467_2022_29879_MOESM1_ESM.pdf]

Supplementary Information
Quantum spins and hybridization in artificially-constructed chains of magnetic adatoms on a superconductor

Eva Liebhaber,[1] Lisa M. Rütten,[1] Gaël Reecht,[1] Jacob F. Steiner,[2] Sebastian Rohlf,[3] Kai Rossnagel,[3,4] Felix von Oppen,[2] and Katharina J. Franke[1]

[1]*Fachbereich Physik, Freie Universität Berlin, 14195 Berlin, Germany*
[2]*Dahlem Center for Complex Quantum Systems and Fachbereich Physik, Freie Universität Berlin, 14195 Berlin, Germany*
[3]*Institut für Experimentelle und Angewandte Physik, Christian-Albrechts-Universität zu Kiel, 24118 Kiel, Germany*
[4]*Ruprecht Haensel Laboratory, Deutsches Elektronen-Synchrotron DESY, 22607 Hamburg, Germany*

SUPPLEMENTARY NOTE 1: THEORETICAL CONSIDERATIONS OF THE DIMER

The YSR states of the dimer are expected to exhibit both a shift and a splitting relative to the monomer. Our measurements show that the shift is comparable to (β) or even considerably larger (α) in magnitude than the splitting. Within a classical-spin model, the shift is of higher order in the coupling between the adatoms and hence generically smaller than the splitting [1]. Specifically, in a tight-binding theory of the YSR dimer assuming classical spins, the shift is controlled by [2]

$$C = \int d\mathbf{r} K(\mathbf{r}+\mathbf{d})\phi^\dagger(\mathbf{r})\phi(\mathbf{r}), \tag{1}$$

while the splitting involves

$$D = \int d\mathbf{r} K(\mathbf{r})\phi^\dagger(\mathbf{r})\phi(\mathbf{r}+\mathbf{d}). \tag{2}$$

Here, $\phi(\mathbf{r})$ is the two-component spinor of the monomer YSR state (centered at $\mathbf{r}=\mathbf{0}$), $\mathbf{d}$ denotes the distance vector between the adatoms constituting the dimer, and $K(\mathbf{r})$ is the exchange coupling between adatom and substrate electrons. When $|\mathbf{d}|$ is larger than the range of the exchange coupling $K(\mathbf{r})$, C and D are of different orders in the small overlap of the YSR wave functions of the dimer. While the shift $C \sim |\phi(\mathbf{d})|^2$ is quadratic in the overlap, the splitting $D \sim |\phi(\mathbf{d})|$ is linear.

In contrast, splitting and shifts are independent of one another within a quantum-spin model. While the splitting originates in the overlap of the YSR wave functions of the monomers, the shift involves the RKKY coupling between the adatom spins. The difference between the classical and quantum models lies in that only in the quantum model, the binding of a quasiparticle is associated with (Kondo-like) screening of the adatom spin. We begin by considering the case of a spin-$\frac{1}{2}$ adatom and comment on higher spins below. Consider first an adatom with a quantum spin. At weak exchange coupling between adatom spin and conduction electrons, the adatom spin remains unscreened and can assume two spin states: $|\Uparrow\rangle$ and $|\Downarrow\rangle$. At stronger exchange coupling, there is a quantum phase transition to a screened state which binds a quasiparticle. In this state, the adatom spin forms a singlet $|\Uparrow\downarrow\rangle - |\Downarrow\uparrow\rangle$ with the quasiparticle spin (spin states $|\uparrow\rangle$ and $|\downarrow\rangle$). In the unscreened state, the adatom spin can orient relative to a neighboring adatom spin and thereby gain RKKY energy. This does not happen in the screened state which is entirely isotropic in spin space. For contrast, consider the effect of quasiparticle binding on a classical spin. While a classical spin can also bind a quasiparticle, the quasiparticle spin is simply oriented opposite to the adatom spin, corresponding to states $|\Uparrow\downarrow\rangle$ and $|\Downarrow\uparrow\rangle$. In these states, the adatom spin remains free to orient relative to adjacent adatom spins, thereby benefitting energetically from the RKKY interaction. Thus, for a quantum spin, the RKKY energy of the dimer is distinctly different for screened and unscreened adatom spins. In contrast, the RKKY energy of a dimer of classical spins is independent of whether the adatom spins bind a quasiparticle or not.

This difference carries over into the energies of YSR resonances. These resonances are excitations between states with and without bound quasiparticles (energies E_{odd} and E_{even}, respectively). The excitation energy equals the energy difference between these states, $E_{\mathrm{YSR}} = |E_{\mathrm{even}} - E_{\mathrm{odd}}|$. In a dimer of quantum spins, the states with and without bound quasiparticles have different RKKY energies and this difference in RKKY energies contributes to the YSR energy of the dimer in a manner that is independent of the hybridization. In contrast, the states with and without bound quasiparticle have the same RKKY energy for a classical spin, and the RKKY interaction cancels out from the YSR energy of a dimer of classical spins (although, of course, the RKKY interaction is nonzero also for classical adatom spins).

The same difference remains operative for higher adatom spins. For higher-spin adatoms, the adatom spin is coupled to multiple conduction-electron channels ($2S$ channels for a spin-S adatom), each of which can bind a quasiparticle [3]. When increasing the exchange coupling within a particular channel, there is a quantum phase transition from a state without bound quasiparticle to a state with bound quasiparticle. For a classical adatom spin, binding or unbinding a quasiparticle leaves the effective impurity spin unchanged. For a quantum spin, each bound quasiparticle reduces the effective spin of the adatom by 1/2. Thus, for a quantum spin, the adatom spin can be screened to any (integer or half-integer) effective spin between zero and S. Exciting one of the YSR resonances changes the effective adatom spin by 1/2, leading to a corresponding change of the RKKY energy of a dimer.

We illustrate the change in RKKY coupling for a dimer of spin-S adatoms, which are each coupled to a single conduction electron channel. In the unscreened state, the RKKY coupling takes the form $J\mathbf{S}_1 \cdot \mathbf{S}_2$. If one of the adatom spins, say $\mathbf{S}_1$ is screened, it effectively acts as a spin-$(S-\frac{1}{2})$ spin $\mathbf{S}_{1,\mathrm{eff}}$, albeit with a prefactor that depends on S. In fact, the projection theorem yields

$$\mathbf{S}_1 \to \frac{2(S+1)}{2S+1}\mathbf{S}_{1,\mathrm{eff}}, \tag{3}$$

so that the RKKY coupling in the presence of screening becomes $J'\mathbf{S}_{1,\mathrm{eff}} \cdot \mathbf{S}_2$ with a modified exchange coupling constant $J' = J[2(S+1)/(2S+1)]$. For ferromagnetic coupling, for instance, this implies that the RKKY energies of the screened and unscreened states differ by

$$\Delta E_{\mathrm{RKKY}} = \frac{S}{2S+1}J. \tag{4}$$

This difference in RKKY energies contributes directly to the energy of YSR resonances of dimers of quantum spins. Interestingly, it approaches a finite constant even in the limit of a large adatom spin.

SUPPLEMENTARY NOTE 2: THEORETICAL CONSIDERATIONS OF THE CHAINS

The results for monomer, dimer, and trimer suggest that both the hybridization and the RKKY coupling of neighboring adatoms in our adatom chains are comparable to or smaller than the superconducting gap. To gain intuition for interpreting the observations of longer chains (but shorter than the scale on which the CDW becomes relevant), we consider a minimal model of $S=\frac{1}{2}$ adatoms coupled to single-site superconductors [3, 4],

$$H = \sum_j \left\{ \Delta\left[c^\dagger_{j,\uparrow}c^\dagger_{j,\downarrow} + \mathrm{h.c.}\right] + \sum_{\sigma\sigma'} c^\dagger_{j,\sigma}[V\delta_{\sigma\sigma'} + K\mathbf{S}_j \cdot \mathbf{s}_{\sigma\sigma'}]c_{j,\sigma'} - t\sum_\sigma \left[c^\dagger_{j,\sigma}c_{j+1,\sigma} + \mathrm{h.c.}\right] + \mathbf{S}_j \cdot \mathrm{J} \cdot \mathbf{S}_{j+1} \right\}, \tag{5}$$

Here, $\mathbf{S}$ denotes the $S=\frac{1}{2}$ adatom spins and J the spin-spin coupling, including both the RKKY interaction (symmetric part of J) and the Dzyaloshinsky-Moriya coupling (antisymmetric part). The exchange coupling between adatom spins and substrate electrons is taken to be isotropic and its strength is denoted by K. The substrate superconductor is reduced to a chain of single-site superconductors (annihilation operator $c_{j,\sigma}$ at site j of the chain) with pairing strength Δ. This description can be effectively thought of as projecting out the quasiparticle continuum and focusing on the subgap quasiparticles induced by the adatom spin. We also include a site energy V, which can be thought of as the strength of potential scattering of substrate electrons by the adatom. Finally, the hybridization of YSR states is captured by including a hopping term of strength t between neighboring single-site superconductors.

First consider the monomer [3]. Its eigenstates can be classified according to the fermion parity of the single-site superconductor. In the even-parity subspace (spanned by the empty and the doubly-occupied superconducting site), the monomer gains pairing energy Δ, but no exchange coupling K. (Both electronic states spanning the even-fermion-parity subspace are spin singlets.) In the odd-parity subspace (spanned by the two singly-occupied states of the superconducting site), the monomer does not gain pairing energy, but benefits from the exchange coupling due to singlet formation between the impurity spin and the substrate electron. Thus, the even-fermion-parity ground state has energy $V-\sqrt{V^2+\Delta^2}$, while the odd-fermion-parity state has energy $V-\frac{3K}{4}$. The energy of the YSR excitation is then given by

$$E_{\mathrm{YSR}} = \sqrt{V^2+\Delta^2} - \frac{3K}{4}. \tag{6}$$

For positive E_{YSR}, the monomer is in the doublet of free-spin ground states

$$|\pm\rangle = |S_z = \pm 1/2\rangle \otimes (u + vc^\dagger_\downarrow c^\dagger_\uparrow)\,|\mathrm{vac}\rangle\,, \tag{7}$$

as the adatom spin remains uncoupled to the substrate electrons. The YSR excitation excites the monomer into the screened-spin state

$$|0\rangle = (|\Uparrow\downarrow\rangle - |\Downarrow\uparrow\rangle)/\sqrt{2}, \tag{8}$$

in which the impurity spin forms a singlet with the substrate-electron spin. For negative E_{YSR}, the monomer is in the screened-spin ground state and the YSR excitation excites the free-spin doublet. In both cases, the energy of the YSR excitation is equal to $|E_{\mathrm{YSR}}|$. Here, double arrows denote the impurity-spin state, whereas single arrows denote the spin state of the substrate electron,

$$|\sigma\rangle = c_\sigma^\dagger |\mathrm{vac}\rangle = \gamma_\sigma^\dagger \left(u + v c_\downarrow^\dagger c_\uparrow^\dagger\right) |\mathrm{vac}\rangle \,. \tag{9}$$

The amplitudes

$$u = \sqrt{\frac{1}{2}\left(1 + \frac{V}{\sqrt{\Delta^2 + V^2}}\right)} \quad , \quad v = \sqrt{\frac{1}{2}\left(1 - \frac{V}{\sqrt{\Delta^2 + V^2}}\right)} \tag{10}$$

denote the conventional electron and hole amplitudes u, v of BCS theory. They also enter the Bogoliubov operators

$$\gamma_\sigma = u c_\sigma + \sigma v c_{\bar{\sigma}}^\dagger \tag{11}$$

of the subgap excitations.

Now consider coupled adatoms [4]. The experimental results suggest that both the RKKY coupling and the YSR hybridization are smaller than the superconducting gap. Here, we thus consider the limit $\Delta, K, V \gg t, J, E_{\mathrm{YSR}}$. We can then restrict the Hilbert space by retaining only the singlets $|0_j\rangle$ (i.e., the screened-spin states) and the doublets $|\pm_j\rangle$ (i.e., the free-spin states) at all sites j of the chain. Within this restricted subspace, the spin-spin interaction J acts only between adjacent sites, which are both in the free-spin state. Moreover, the hybridization t changes the fermion parity of the two participating sites, thereby enabling two processes. Neighboring free-spin and screened-spin sites can exchange positions with effective amplitude $\tilde{t} = t(u^2 - v^2)/2 = tV/2\sqrt{\Delta^2 + V^2}$. Similarly, two adjacent free-spin sites with opposite spin can be converted into two screened sites (or vice versa) with amplitude $\tilde{\Delta} = tuv = t\Delta/2\sqrt{\Delta^2 + V^2}$.

We use exact diagonalization via the Lanczos scheme (with up to 300 states in the excited state sectors) to study the local single-particle spectral function

$$A_j(E) = \frac{\kappa}{\pi} \sum_{\sigma,\lambda} \left[\frac{\left| \langle\lambda| c_{j,\sigma}^\dagger |\mathrm{g.s.}\rangle \right|^2}{\kappa^2 + (E - E_\lambda + E_{\mathrm{g.s.}})^2} + \frac{\left| \langle\lambda| c_{j,\sigma} |\mathrm{g.s.}\rangle \right|^2}{\kappa^2 + (E + E_\lambda - E_{\mathrm{g.s.}})^2} \right]. \tag{12}$$

The local single-particle spectral function is expected to describe tunneling experiments using superconducting tips in the limit of weak tip-substrate tunneling. We note that our numerical results are restricted in a variety of ways relative to the experimental system. They are limited to spin-$\frac{1}{2}$ impurities, do not resolve the local YSR wave functions or accommodate specifics of the substrate due to the single-site approximation for the superconductor, are restricted to zero temperature, and involve a phenomenological broadening κ. The assumption of zero temperature is restrictive since we deduce a spin-spin interaction J which is comparable to temperature. Consequently, one expects that excited states of the spin chain, involving for instance domain walls within a ferromagnetically ordered chain can play a significant role in the experimental chains.

Within the quantum-spin model, energy bands and van Hove singularities tend to form in particular for ferromagnetic spin ordering. In this case, the tunneling electron changes the local spin state (by screening or unscreening the local spin), creating a mobile impurity in the spin chain. In the ferromagnetic phase, this impurity can hop to neighboring sites and effectively forms a single-particle band with van Hove singularities. Supplementary Figure 1a shows the formation of the band as the number of adatoms N is increased, as seen in the local spectral function. The lower panel exhibits the local spectral function resolved all along a $N = 10$ chain. Qualitatively consistent with the experimental observation, one observes that there are incipient van Hove peaks at the two band edges. Presumably, these simulations assume better energy resolution than available in experiment, which overemphasizes the bending of the van Hove singularity along the chain due to the detailed node structure of the individual wave functions. This structure may be further reduced when including thermally excited spin states. The upper panel shows how the van Hove singularity emerges with increasing N, again qualitatively consistent with our experimental observations.

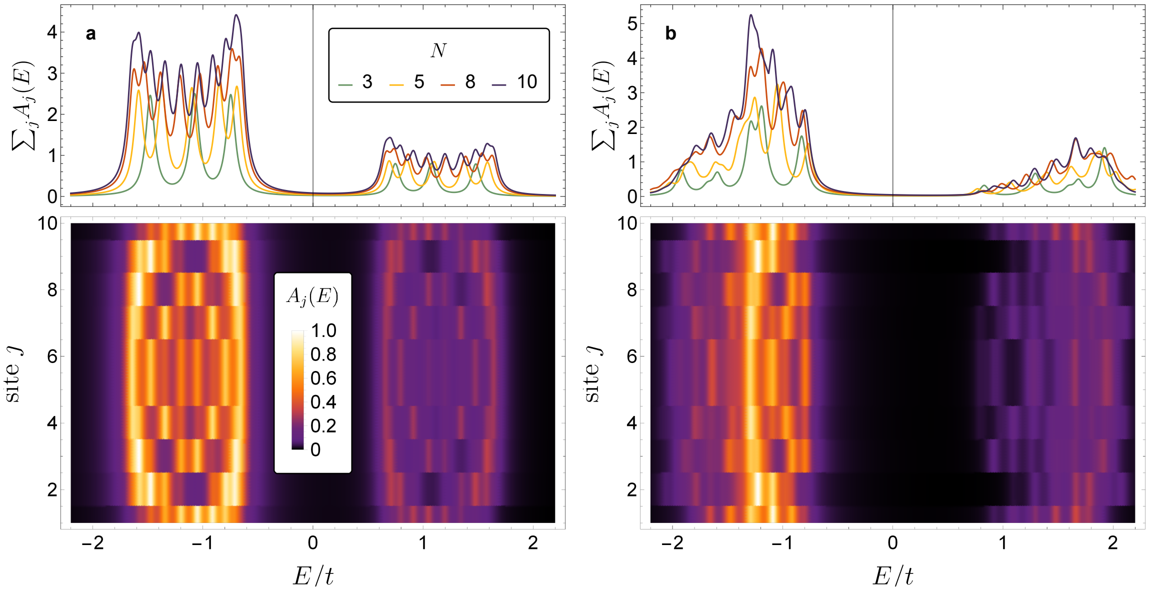

Supplementary Figure 1. **Spectral functions of unscreened spin-$\frac{1}{2}$ adatom chains.** Local single-particle spectral density for a chain of spin-$\frac{1}{2}$ adatoms as modeled by the Hamiltonian in Supplementary Eq. (5). Top panels show single-particle spectral function integrated over the entire chain for different chain lengths N. Bottom panels exhibit spatially resolved spectral function for $N = 10$. **a** Ferromagnetic phase (fully unscreened phase) with $E_{\mathrm{YSR}} = t$ $J = -0.3t$ and a miniscule magnetic field $B_z = 0.001t$. **b** Antiferromagnetic phase (fully unscreened phase) with $E_{\mathrm{YSR}} = t$, $J = 0.3t$. Other parameters applicable to all panels: $V = -0.6\Delta$ (chosen such that $|v|^2 \simeq 3|u|^2$, similar to the experimental peak heights in Fig. 2, main text) and $\kappa = 0.05t$.

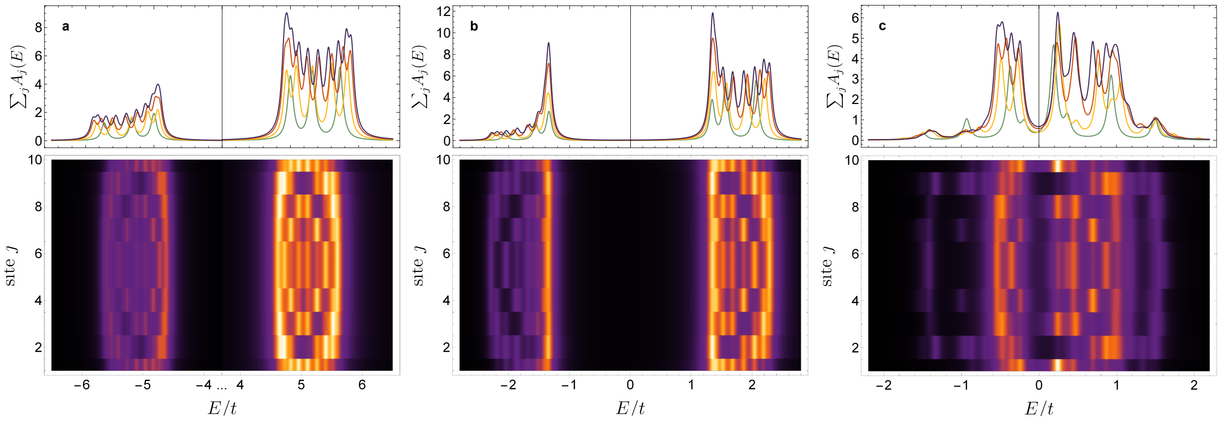

Supplementary Figure 2. **Spectral functions of (partially) screened spin-$\frac{1}{2}$ adatom chains.** Local single-particle spectral density for a chain of spin-$\frac{1}{2}$ adatoms as modeled by the Hamiltonian in Supplementary Eq. (5). Top panels show single-particle spectral function integrated over the entire chain for different chain lengths N. Bottom panels exhibit spatially resolved spectral function for $N = 10$. **a** Fully screened phase with $E_{\mathrm{YSR}} = -5t$. **b** Partially screened chain with incipient singlet superconducting correlations with $E_{\mathrm{YSR}} = -1.5t$. **c** Singlet superconducting phase with $E_{\mathrm{YSR}} = 0$. Other parameters applicable to all panels: $V = -0.6\Delta$ (chosen such that $|v|^2 \simeq 3|u|^2$, similar to the experimental peak heights in Fig. 2, main text), $J = 0$, and $\kappa = 0.05t$. Color code in top panels as in Supplementary Fig. 1.

For contrast, Supplementary Fig. 1b shows corresponding results for antiferromagnetic RKKY coupling. Unlike for ferromagnetic spin ordering, the excitation spectrum is not single-particle-like and does not exhibit pairs of van Hove singularities for any chain length. Single-particle bands and the emergence of van Hove singularities with increasing N are also observed when all spins are screened, see Supplementary Fig. 2a. However, the experimental results indicate that the current system does not realize this limit (unscreened δ resonance as well as partially screened α band). Panels b and c of Supplementary Fig. 2 show corresponding results for monomer YSR energies such that there is only partial screening. In this case, the single-particle spectral function exhibits a pair of peaks which are located at symmetric

energies relative to the Fermi energy. These peaks are BCS coherence peaks induced by the singlet superconducting correlations $\tilde{\Delta}$. For unscreened δ resonance, this phase should not be realized in the experimental system, since ferromagnetic order suppresses the underlying singlet correlations. We also do not observe the well-developed gap around the Fermi energy which exists in this phase.

As discussed in the main text, we assume that the δ resonance is likely unscreened. However, this assignment (based on analyzing shifts of the YSR energy with adsorption position relative to the CDW) is less definitive than the ones for α-γ. We briefly comment on this issue in light of the observed band formation. When all YSR states are in the screened state in the monomer, the adatom spin is effectively fully screened in the monomer. Due to the quantum phase transition for the α resonance, we would then conclude that the adatom chains can effectively be viewed as partially filled spin-$\frac{1}{2}$ chain. In this case, there is a substantial region in the phase diagram in which the system is in a spin-singlet superconducting phase (in addition to the ferromagnetic phase discussed above) [4]. As seen in panels b and c of Supplementary Fig. 2, the coherence peaks of this singlet superconductor naturally provide a single dominant peak which might be consistent with our observations of the α band. Indeed, as discussed in the main text, it is possible (though less likely) that this resonance leads to symmetric peaks about the Fermi energy. However, an interpretation of this observation in terms of coherence peaks of a spin-singlet superconductor seems unlikely since we do not observe the associated gap around the Fermi energy. Note in particular that according to the top panels, the zero-temperature spectral function does not exhibit any structure within this gap for any chain length N. Moreover, the observation of two peaks for the β band is much less natural in such a spin-singlet superconductor, as its excitation spectrum does not exhibit single-particle character. Based on these considerations, we conclude that the observations on adatom chains support our identification of the δ resonance as unscreened.

SUPPLEMENTARY NOTE 3: ADSORPTION SITES AND INCOMMENSURATE CDW

Fe atoms deposited at low temperature on the clean $NbSe_2$ surface adsorb in two distinct sites. They can be distinguished by their different apparent height (Supplementary Fig. 3a) and identified as sitting in the two different hollow sites of the terminating Se layer, which differ by the presence (metal-centered, MC) or absence (hollow-centered, HC) of a Nb atom underneath. The YSR states from the Fe atoms in the different adsorption sites differ substantially [5]. Here, we investigate and build up chains from Fe atoms on the HC sites only.

The incommensurate nature of the CDW with a periodicity of $\gtrsim 3a \times 3a$ (see white arrow in Supplementary Fig. 3a) is reflected in an additional modulation to the atomic corrugation in the STM images. When the maximum of the CDW is located on a hollow site of the Se layer (hollow-centered, HC), the topographic pattern appears with a three-petaled shape (yellow area in Supplementary Fig. 3a and Supplementary Fig. 3b). In contrast, when the CDW maximum lies on top of a Se atom (chalcogen-centered, CC), the STM image is petalless (red area in Supplementary Fig. 3a and Supplementary Fig. 3c). Due to the incommensurability of the CDW, the patterns smoothly transform into one another.

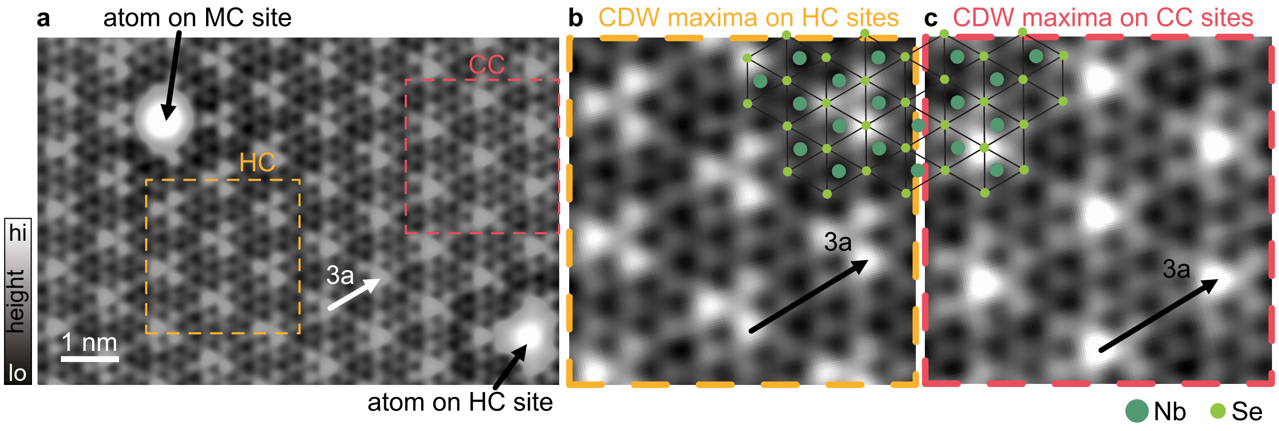

Supplementary Figure 3. **Adsorption sites on the incommensurate CDW. a** Atomic resolution topography (constant-current mode, set point: 100 pA, 10 mV). **b** and **c** are zooms into the areas marked in **a**. Arrows indicate one CDW period ($\approx 3a$). The overlaid grid shows the terminating Se-layer (bright green) and the Nb layer beneath (dark green).

SUPPLEMENTARY NOTE 4: KONDO EFFECT

In the main text, we show that the splitting and shift of YSR states of the Fe adatoms due to RKKY interactions cannot be explained with a classical-spin model, but instead requires a quantum mechanical description. Another expression of the quantum nature of the Fe adatom's spin is found when inspecting the spectroscopic signature in the normal state of the substrate. Supplementary Figure 4 shows a zero-bias peak measured on an Fe atom sitting on a hollow site of the Se lattice at a temperature of 8 K. This resonance reveals a Kondo resonance as a fingerprint of a quantum spin on the surface.

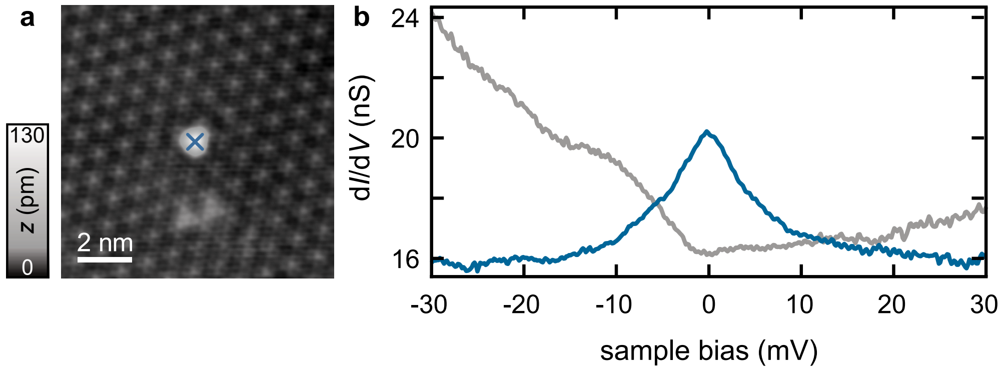

Supplementary Figure 4. **Kondo effect on single Fe adatom.** **a** Topography showing a single Fe atom (constant-current mode, set point: 200 pA, 4 mV) recorded with a Pb tip. **b** Differential conductance spectra recorded on the atom (blue) and on the bare $NbSe_2$ (gray) at $T = 8$ K. Feedback was opened at 500 pA, 30 mV and a modulation of 0.5 mV was used.

SUPPLEMENTARY NOTE 5: ATOM MANIPULATION USING A SUPERCONDUCTING NB TIP

All measurements were performed with a superconducting Nb tip fabricated by indenting a NbTi-tip into a superconducting Nb sample until a sharp stable tip apex exhibiting the full Nb gap of $\Delta \approx 1.55$ meV is achieved. To construct the adatom chains, controlled manipulation of the Fe atoms is required. The atoms could be positioned in a very precise manner after placing the STM tip in their close vicinity and dragging them across the surface, while applying small bias voltages (∼mV) and currents in the nA-regime (exact values depend on the tip apex).

SUPPLEMENTARY NOTE 6: DECONVOLUTION AND FITTING PROCEDURE

Due to the superconducting properties of the STM tip, the dI/dV spectra are a convolution of the density of states of tip and substrate. To extract the density of states of the substrate, we numerically deconvolve the spectra as described in the supplementary information to Ref. [5]. Deconvolved spectra of the monomer, dimer and trimer (original spectra in Fig. 2a,c,e of the main manuscript) are shown in Supplementary Fig. 5a. To determine the shift and split of the α- and β-derived YSR states in the dimer and trimer structure, we symmetrized the deconvolved spectra with respect to zero energy and then fitted them with the appropriate number of Gaussian peaks using the following equation:

$$\mathrm{DOS}(E) = D_0 + \sum_{i=1}^{N} \left(A_i \, e^{-(E \pm E_{\alpha_i})^2/(2\sigma^2)} + B_i \, e^{-(E \pm E_{\beta_i})^2/(2\sigma^2)} \right). \qquad (13)$$

Here, D_0 is an intensity offset, A_i (B_i) are the (symmetric) amplitudes of the α- (β-)derived resonances and σ is the width of the Gaussian peaks. The number of split YSR states depends on the number of atoms N in the hybridized structure, being two states in the dimer ($\alpha^{\mathrm{d0,d1}}$ and $\beta^{\mathrm{d0,d1}}$) and three YSR states in the trimer ($\alpha^{\mathrm{t0,t1,t2}}$ and $\beta^{\mathrm{t0,t1,t2}}$). As shown in the main manuscript, the YSR states exhibit strong spatial intensity variations, with some peaks being hardly visible in some spectra. The most reliable determination of all peak positions thus follows from a detailed analysis of a set of spectra from the line profiles in Fig. 2b,d,f of the main text. Several spectra were fitted and the fit

	$\lvert D_\alpha(\mu\text{eV})\rvert$	$\lvert D_\beta(\mu\text{eV})\rvert$	$C_\alpha(\mu\text{eV})$	$C_\beta(\mu\text{eV})$
dimer	69 ± 33	173 ± 33	$+184 \pm 29$	-160 ± 29
trimer	388 ± 62	286 ± 79	$+14 \pm 33$	-201 ± 36

Supplementary Table I. **Split and shift of YSR states.** Results for the splits $D_{\alpha,\beta}$ and shifts $C_{\alpha,\beta}$ (relative to the monomer) of the hybrid α- and β-states of the dimer and trimer obtained from Fig. 3e main part.

results of the YSR energies were averaged. The error bars of the YSR energies were determined from the standard deviation of the fit, the error margin of the energy gap of the tip, the modulation voltage of the lock-in, and the sampling interval of the data used for the deconvolution.

Figure 3e (main part) compiles the resulting peak positions in the monomer, dimer and trimer. Supplementary Table I summarizes the energy splittings $D_{\alpha,\beta}$ and shifts $C_{\alpha,\beta}$ in the hybridized structures relative to the monomer.

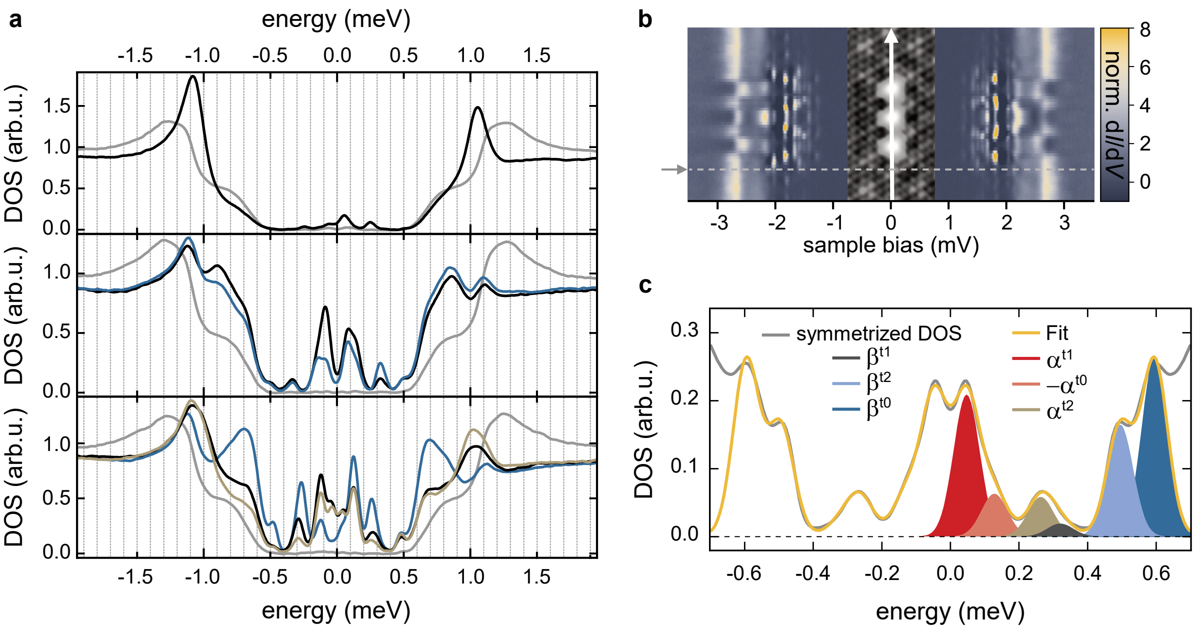

Supplementary Figure 5. **Illustration of deconvolution procedure.** **a** Deconvolved data of Fig. 2a,c,e. For the deconvolution $\Delta_{\text{tip}} = 1.55\,\text{meV}$ and a depairing factor of $\Gamma = 5\,\mu\text{eV}$ were used. **b** Data reproduced from Fig. 2f. The gray dashed line marks the position of the spectrum that is deconvolved and symmetrized in **c** (gray line). The fit (yellow) and the individual Gaussian peaks (only at positive energy) are shown in red and blue colors for the α- and β-derived states, respectively.

An example of a fitted trace of a trimer is shown in Supplementary Fig. 5c (position of the original spectrum marked by the arrow and dashed line in Supplementary Fig. 5b). The gray trace is the symmetrized density of states. The fit according to Supplementary Eq. 13 is shown in yellow and the individual Gaussian peaks are shown (for positive energies only) in color. The fit yields reliable values for α^{t0}, α^{t1} and β^{t2}, as they are well separated peaks. In contrast, as discussed in the main text, α^{t2} and β^{t1} strongly overlap leading to one broad resonance (full width half maximum of $\approx 100\,\mu\text{eV}$). The peak corresponding to β^{t0} is very close to the quasiparticle coherence peaks. For these reasons, we added additional error margins of $\pm 50\,\mu\text{eV}$ to the latter three resonances.

SUPPLEMENTARY NOTE 7: INFLUENCE OF THE FE ATOMS TO THE CDW

As described in the main manuscript, an 11-atom chain can be built in such a way that all Fe atoms sit on maxima of the CDW. This contrasts the smooth variation of the CDW and indicates that the Fe atoms may help to lock the phase of the CDW. Upon further extension of the chain, the locking is not operable anymore and the atoms cannot sit on the CDW maxima. From this length on, the smoothly varying phase along the atomic lattice leads to shifts of the YSR band structure. Importantly, during the manipulation and extension of the chain up to a chain length of

$N = 30$ atoms, the CDW did not change abruptly. In contrast, upon attachment of the 31st atom, an abrupt change occurred in the CDW (compare Supplementary Fig. 6a and b). This change is most clearly expressed in a change of the dI/dV spectra. While the 30-atom chain exhibits the smooth variations of van Hove singularities toward the chain's terminations, the 31-atom chain shows two distinct areas of YSR bands. Close inspection of the CDW phase reveals that the atoms in the area marked by a white arrow are now located on minima of the CDW (Supplementary Fig. 6b,d). These observations suggest that the Fe atoms favor adsorption sites on the maxima or minima of the CDW and thus push the CDW into the respective phase. However, when too much energy is stored in the locked CDW, stress is released by an abrupt switching of the CDW.

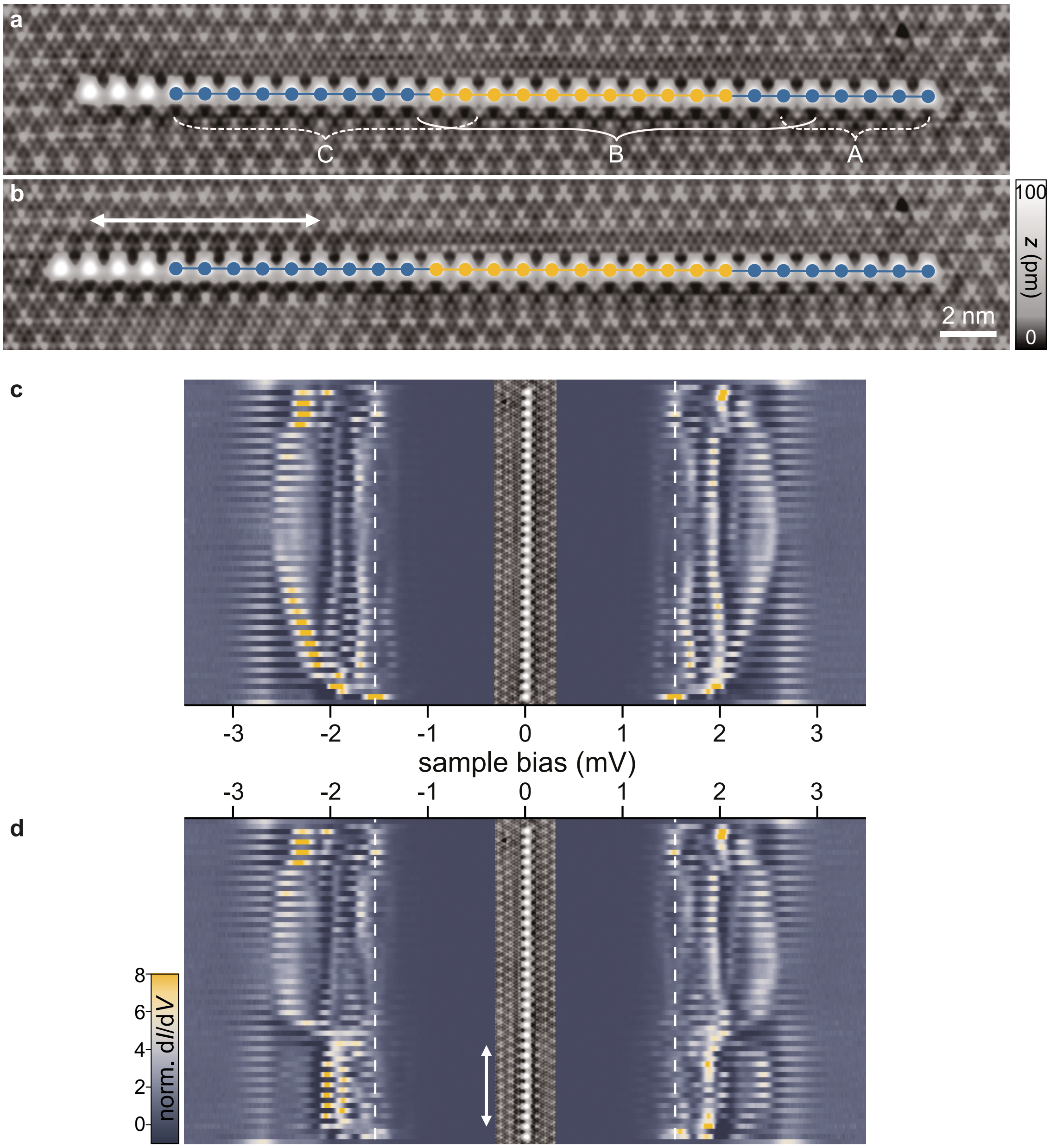

Supplementary Figure 6. **Switching of CDW and YSR bands across different domains.** **a,b** Constant-current topography of the 30-atom chain **a** and 31-atom chain **b** recorded at a set point of 100 pA, 10 mV. The former 11-atom (27-atom) chains are indicated in yellow (blue). The white arrow marks the section of the 31-atom chain where atoms are located on a CDW minimum. **c,d** Line profiles of normalized dI/dV spectra recorded along the 30-atom chain **c** and 31-atom chain **d** (constant-height mode, feedback opened at 700 pA, 5 mV with a modulation of 15 µV). The arrow marks the same section as in **b**. Dashed lines indicate the tip gap.

SUPPLEMENTARY NOTE 8: ADDITIONAL $\mathrm{d}I/\mathrm{d}V$ MAPS

In the main manuscript, we have presented selected $\mathrm{d}I/\mathrm{d}V$ maps of monomers, dimer, trimers and an extended 27-atom chain, which helped identifying the nature of the YSR states. This section provides additional data, which corroborate the assignments in the main text.

Supplementary Figure 7a-c complement the $\mathrm{d}I/\mathrm{d}V$ maps presented in Fig. 3 of the main manuscript with the opposite bias polarity. Additionally, Supplementary Fig. 7d,e show $\mathrm{d}I/\mathrm{d}V$ maps of the thermally activated α-derived resonances at $eV_{\mathrm{YSR}} = \mp|\Delta_{\mathrm{tip}} - E_{\mathrm{YSR}}|$. These data support the identification of the α- and β-derived states by their distinct symmetries and presence/absence of nodal planes. In particular, the assignment of both α states of the dimer to lie at the other bias polarity becomes clear when inspecting the thermal maps. The patterns of the monomer, dimer, and trimer share several features, which help in the identification of the origin of the β-derived states. For instance, oscillations along the white arrows in Supplementary Fig. 7a are the same along the arrow in Supplementary Fig. 7b, and thus indicate the β nature of the dimer state at -2.05 mV. The scattering patterns encircled by white dashed lines in the dimer of Supplementary Fig. 7b, can be found in the trimer again in Supplementary Fig. 7c.

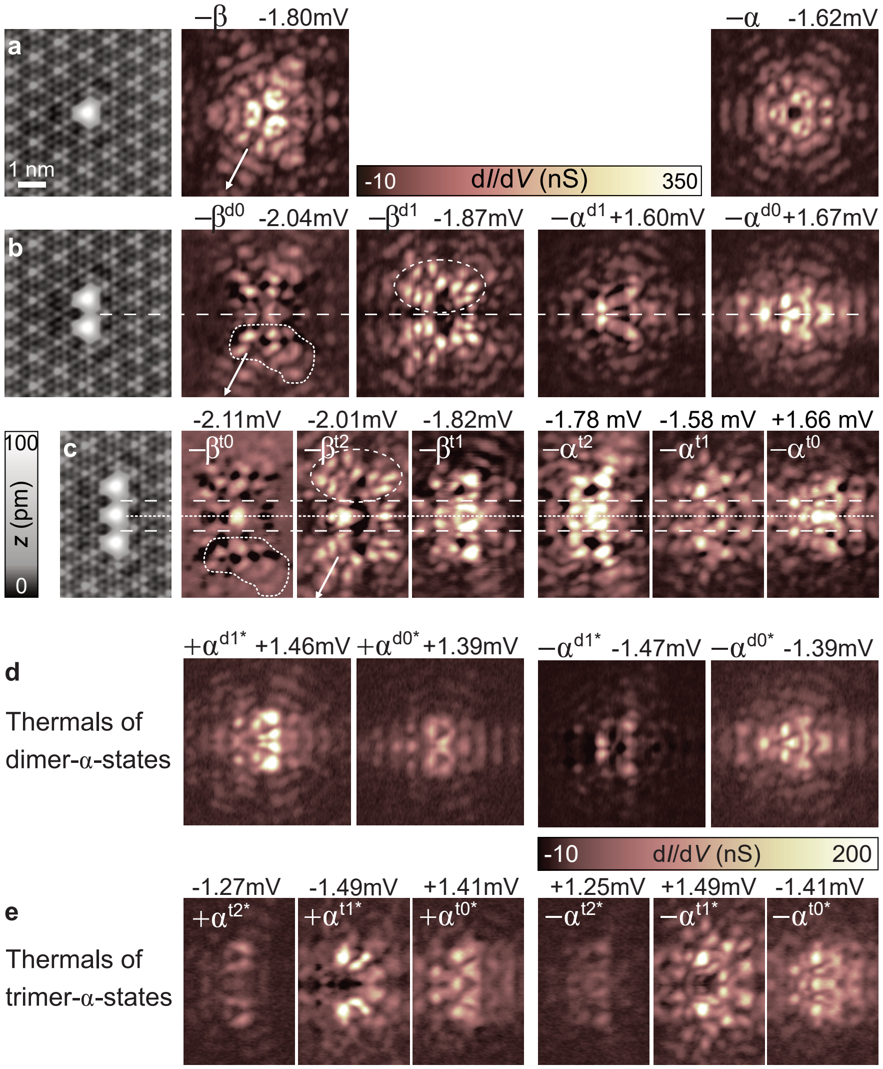

Supplementary Figure 7. **YSR wave functions of monomer, dimer and trimer.** Complementary data of Fig. 3 in the main part. **a-c** STM topographies (constant-current mode with set point 100 pA, 10 mV) of one to three Fe atoms with spacing of $3a$ in the left. Corresponding constant-contour $\mathrm{d}I/\mathrm{d}V$ maps of the (hybridized) YSR states in the monomer (top row), dimer (middle row) and trimer (bottom row). Bias voltages are given above each panel ($V_{\mathrm{YSR}} = \pm|\Delta_{\mathrm{tip}} + E_{\mathrm{YSR}}|/e$ with $\Delta_{\mathrm{tip}} \approx 1.55$ meV). Constant-contour feedback set point is 250 pA, 5 mV and the modulation is 15 µV. **d,e** Constant-contour maps (same parameters as in **a-c**) of the thermally activated α-states of the dimer **d** and the trimer **e**.

Supplementary Figures 8 and 9 show additional dI/dV maps at various energies for chain lengths between 4 and 11 atoms. The maps reflect the character of the hybrid wave functions and their intensity distributions along shorter chains and further highlight the extended nature of the YSR bands for increasing chain length ($N > 5$). We can identify the origin of the bands at larger chain length by structural elements common to the single atom, short and long chains. For example, we find signatures of the β-derived state within the coherence peaks always around $\gtrsim 2.15\,\mathrm{mV}$ and around 1.95 mV as indicated by the blue arrows. States deep within the gap arise from α-states as suggested by comparing the shapes to the α-trimer states (Figs. 3c main part and Supplementary Fig. 7).

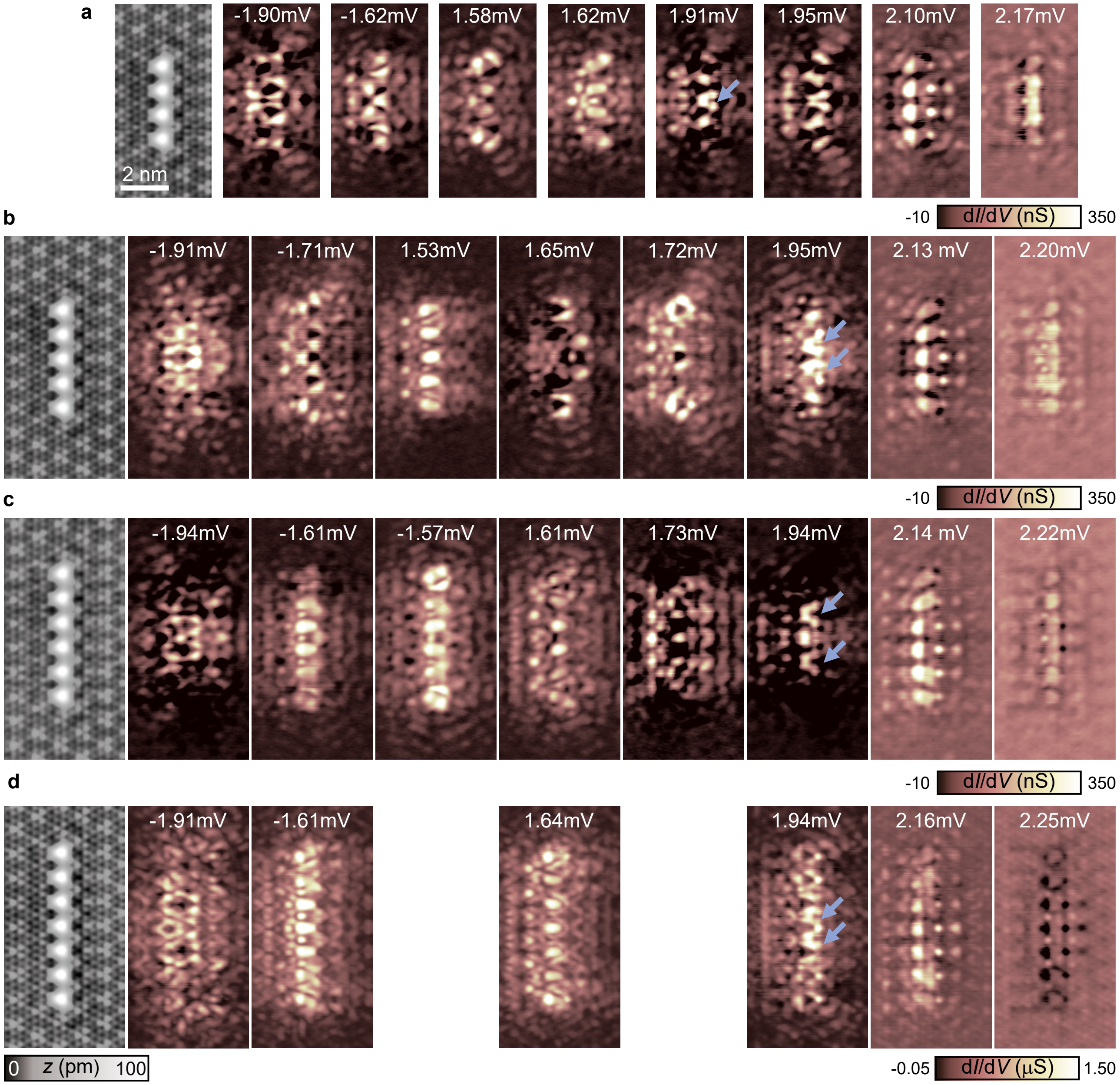

Supplementary Figure 8. **YSR wave functions in chains of $N = 4 - 7$ Fe atoms.** Complementary data to Fig. 4a-d of the main manuscript. **a-d** STM topographies (constant-current mode with set point 100 pA, 10 mV) of the 4-, 5-, 6- and 7-atom chains with spacing of $3a$ on the left. Corresponding constant-contour dI/dV maps at selected bias voltages (values indicated in the panels) on the right. Constant-contour feedback was opened at 250 pA, 5 mV (**a-c**) or 700 pA, 5 mV (**d**) and a modulation of 15 µV was used ($\Delta_{\mathrm{tip}} \approx 1.55\,\mathrm{meV}$).

As mentioned in the main text there is a zero-energy end state ($\Delta_{\text{tip}} \approx 1.55\,\text{mV}$) in the 10-atom chain. However, as can be inferred from the line spectra in Fig. 4g,h in the main paper, this state is shifted to slightly higher energies at the 11-atom chain (1.58 meV). dI/dV maps shown in Supplementary Fig. 9 sustain this interpretation.

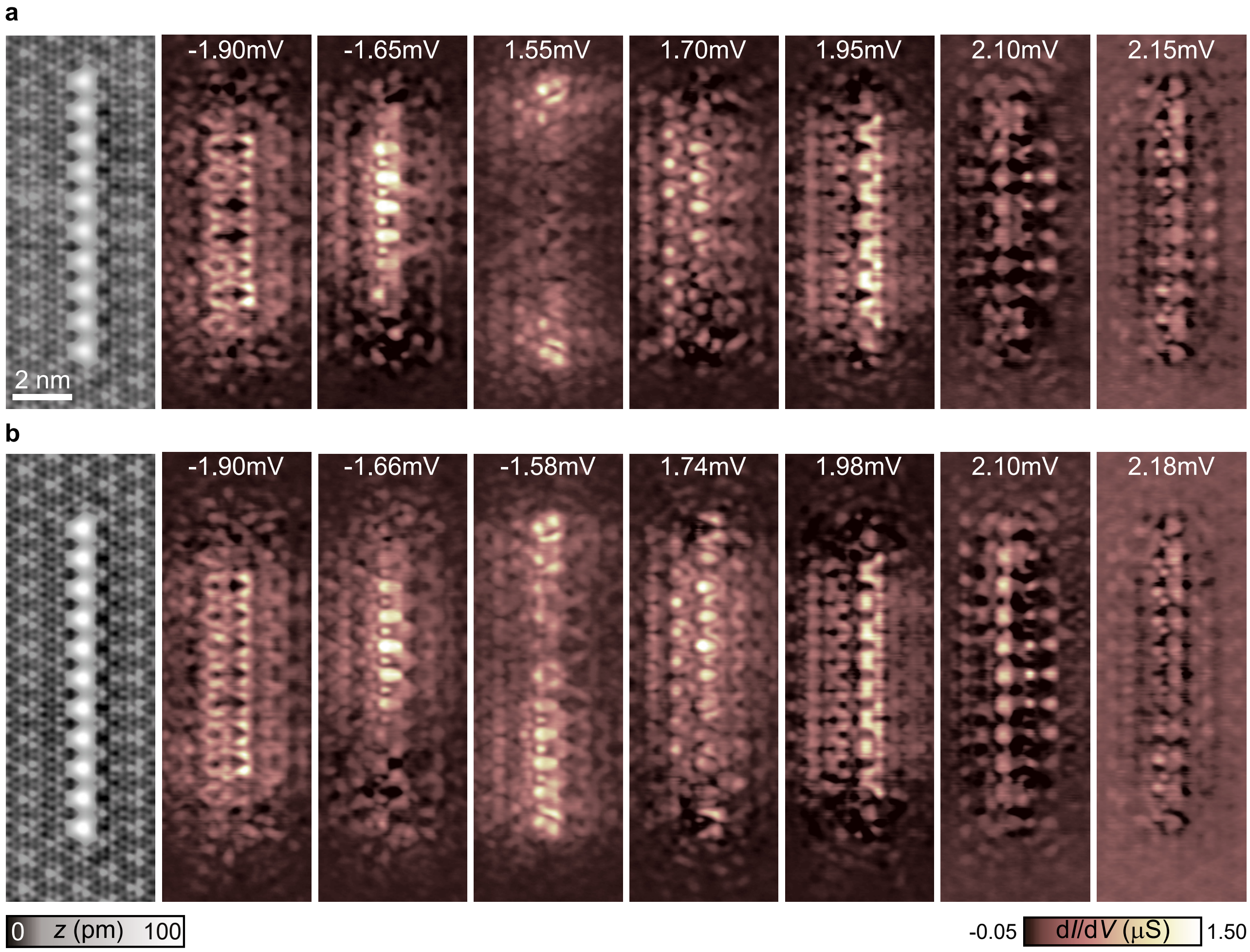

Supplementary Figure 9. **YSR wave functions in chains of $N = 10$ and $N = 11$ Fe atoms.** Complementary data to Fig. 4g,h of the main manuscript. **a,b** STM topographies (constant-current mode with set point 100 pA, 10 mV) of the 10- and 11-atom chains with spacing of $3a$ in the left. Corresponding constant-contour dI/dV maps at selected bias voltages (values indicated in the panels) on the right. Constant-contour feedback was opened at 700 pA, 5 mV and a modulation of 15 µV was used ($\Delta_{\text{tip}} \approx 1.55\,\text{meV}$).

In the main text, we discussed the band bending in a 27-atom chain due to the CDW. Supplementary Figures 10 and 11 show a set of $\mathrm{d}I/\mathrm{d}V$ maps, reflecting the energetic and spatial evolution of the YSR bands. These allow to track the evolution of the bands along the chain. For instance, the highest-energy feature in the chain appears at 2.50 mV in the center of the chain, but at 2.26 mV at the chain's terminations. The van Hove singularity of the β-derived band at ~ 1.95 mV appears at the same energy almost along the entire chain (map at 1.94 mV). Similarly, the van Hove singularity of the α-derived band at -1.70 mV exhibits a small shift towards zero energy only at the terminations (see map at 1.54 mV).

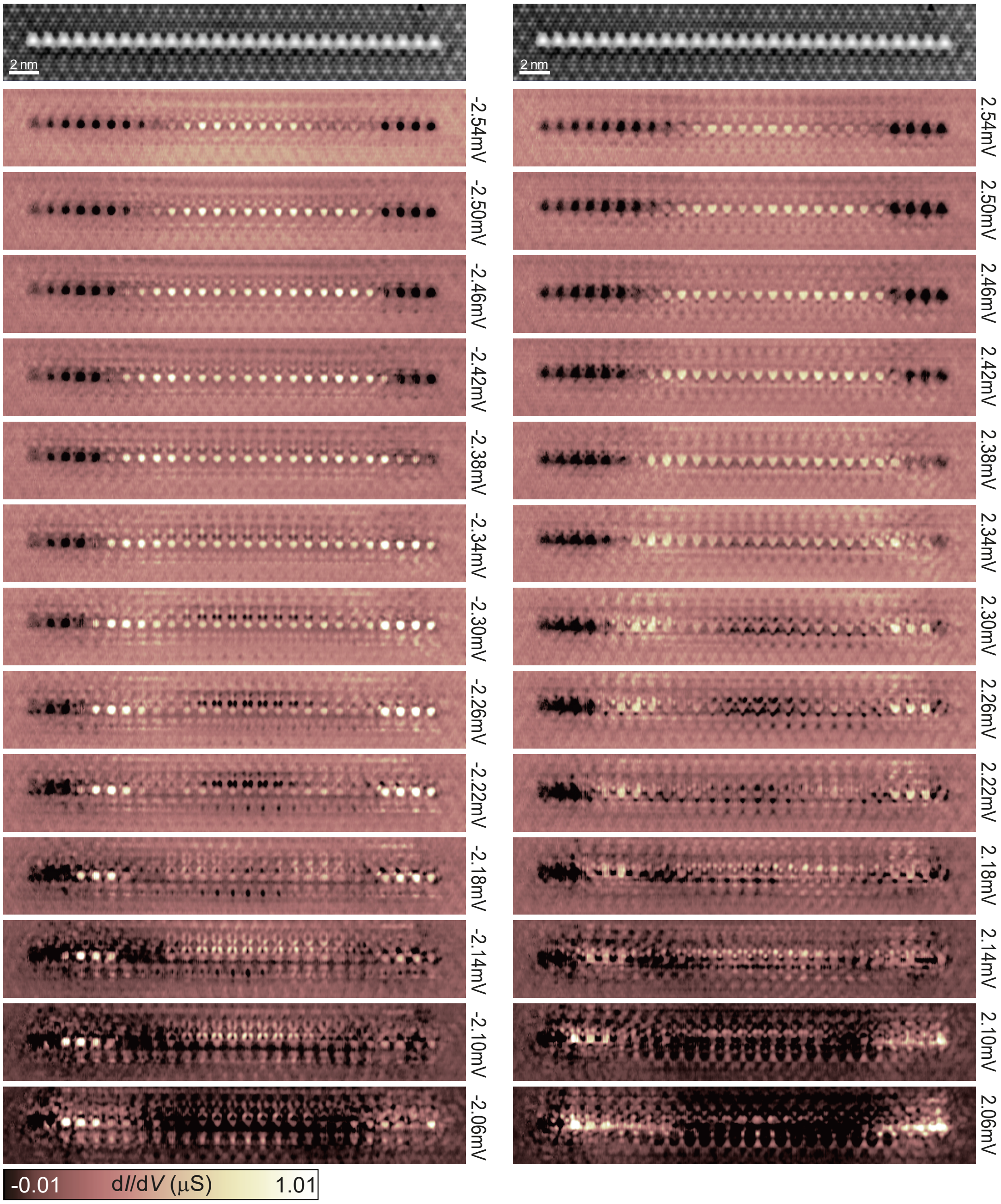

Supplementary Figure 10. **YSR wave functions of 27-atom chain (part I).** Complementary data to Fig. 5 in the main part. Constant-contour $\mathrm{d}I/\mathrm{d}V$ maps recorded at the voltages given next to each panel ($\Delta_{\mathrm{tip}} \approx 1.55$ meV). Feedback was opened at 700 pA, 5 mV and a modulation of 15 µV was used. Topography can be found in the top (set point: 100 pA, 10 mV).

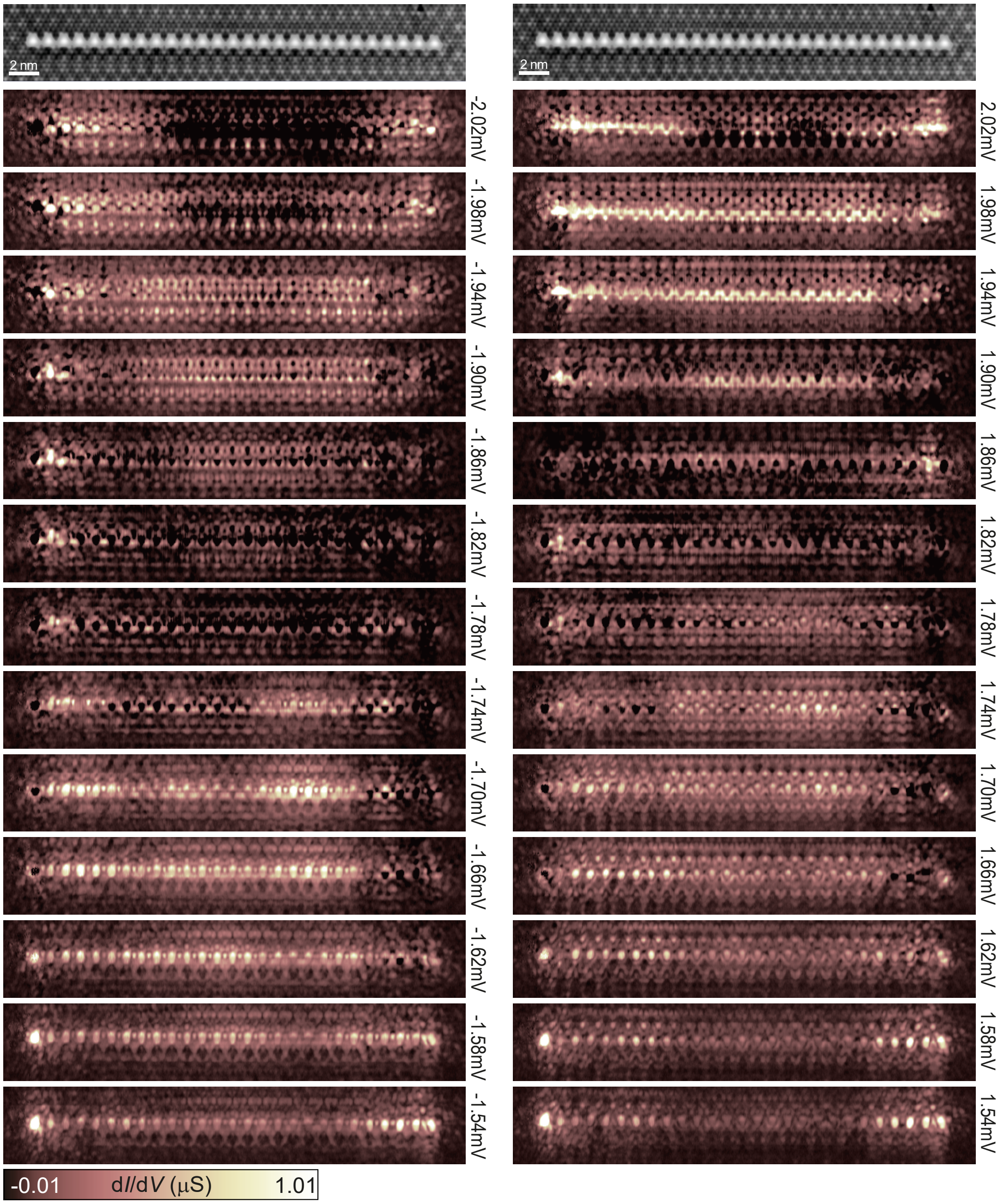

Supplementary Figure 11. **YSR wave functions of 27-atom chain (part II).** Complementary data to Fig. 5 in the main part. Constant-contour $\mathrm{d}I/\mathrm{d}V$ maps recorded at the voltages given next to each panel ($\Delta_{\mathrm{tip}} \approx 1.55\,\mathrm{meV}$). Feedback was opened at $700\,\mathrm{pA}, 5\,\mathrm{mV}$ and a modulation of $15\,\mu\mathrm{V}$ was used. Topography can be found in the top (set point: $100\,\mathrm{pA}, 10\,\mathrm{mV}$).

Supplementary Figure 12 shows dI/dV maps of the 51-atom chain at selected energies, which highlight the localization of the YSR bands in the different regions. In particular, region A shows the down-shifted bands at the chain's termination, region B shows the bands of a chain with Fe atoms sitting on CDW maxima, region C represents the abrupt transition between the Fe atoms located on maxima (region B) and minima (region D) of the CDW, region E shows again band shifts at the chain termination.

Removal of three Fe atoms in region C leads to two separate chains (as the CDW starts to distort also on the left end, we also removed one atom there). dI/dV maps taken at the same energies as for the uninterrupted chain show very similar intensity distributions (compare Supplementary Fig. 12 and Fig. 13). Regions A, B and D, E of the complete 51-atom chain can thus be interpreted as two non-interacting sub-chains.

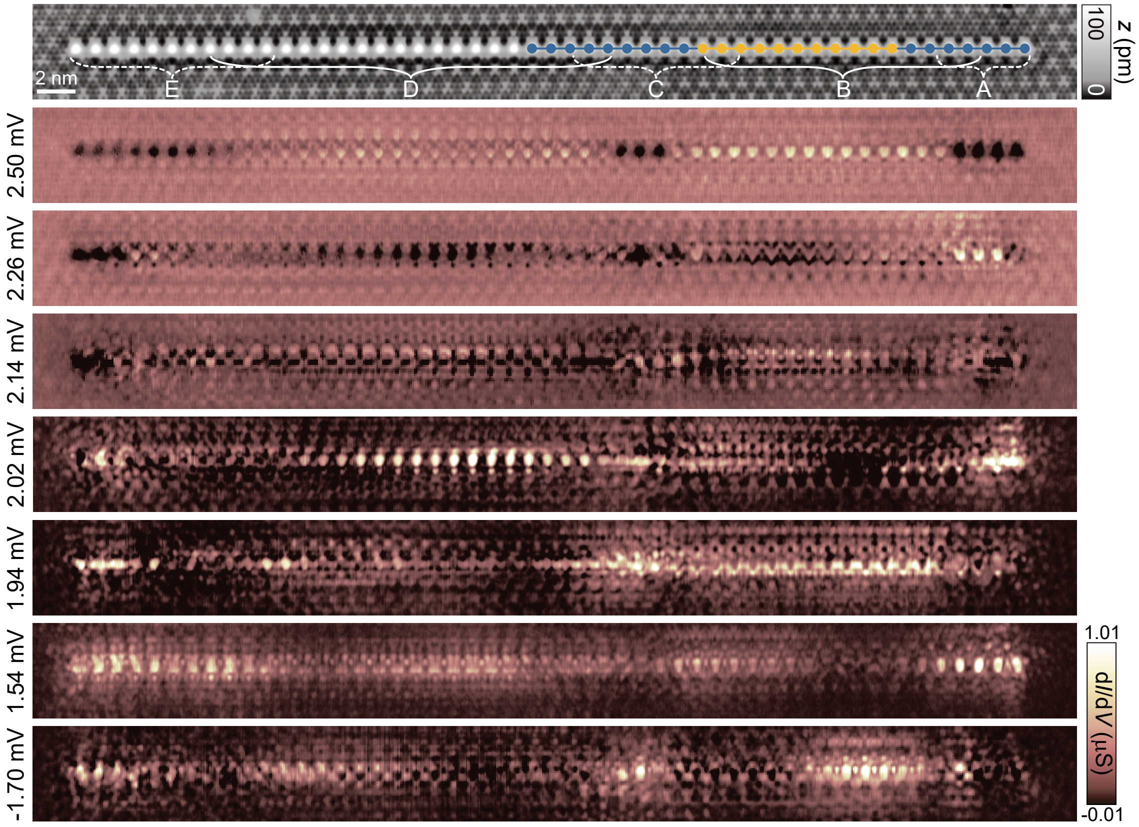

Supplementary Figure 12. **YSR wave functions of 51-atom chain across different domains of the CDW.** Selected constant-contour dI/dV maps recorded on the 51-atom chain (Fig. 6 in the main part) at the voltages given at the left to each panel ($\Delta_{\mathrm{tip}} \approx 1.55$ meV). Feedback was opened at 700 pA, 5 mV and a modulation of 15 μV was used. Topography can be found in the top (set point: 100 pA, 10 mV). The former 11- (27-) atom chain is indicated in yellow (blue).

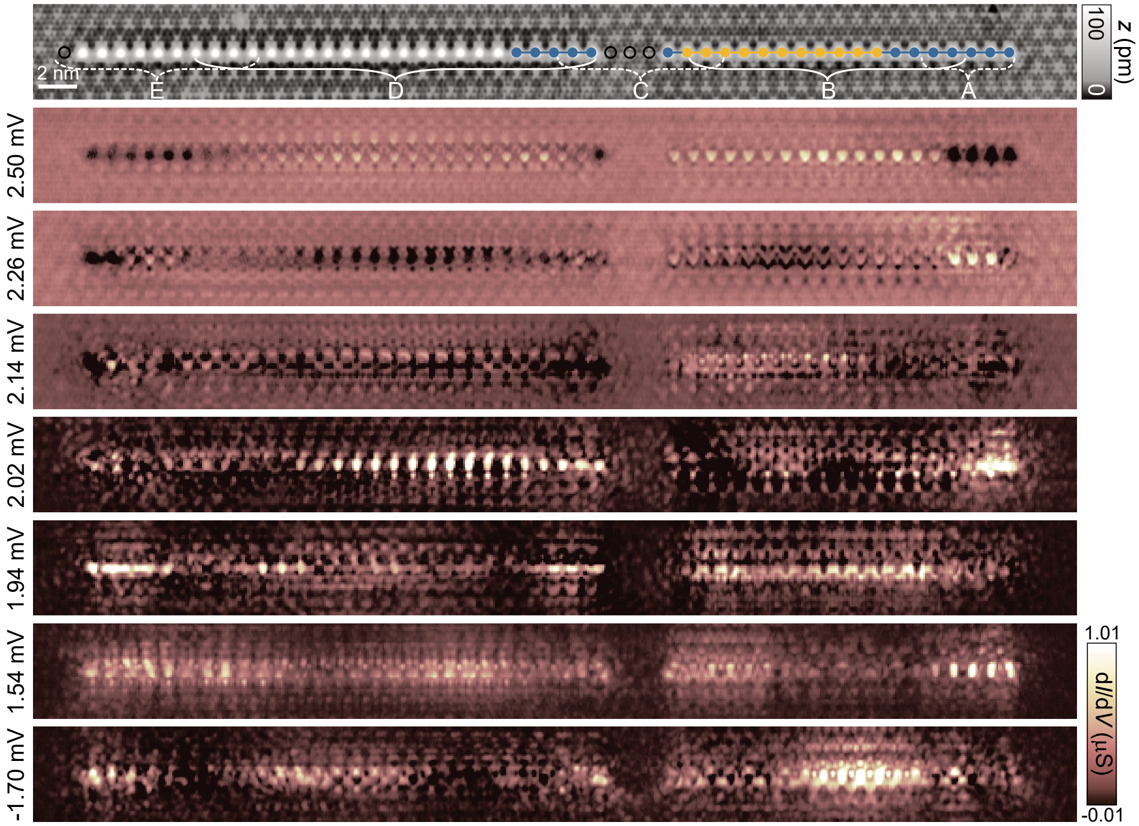

Supplementary Figure 13. **YSR wave functions of two chains on different domains of the CDW.** Selected constant-contour dI/dV maps recorded on the former 51-atom chain (Fig. 6 in the main part) after removal of a few atoms (black circles). Voltages given on the left to each panel ($\Delta_{\mathrm{tip}} \approx 1.55\,\mathrm{meV}$). Data in the different sections are very similar to the 51-atom chain. Feedback was opened at 700 pA, 5 mV and a modulation of 15 µV was used. Topography can be found in the top (set point: 100 pA, 10 mV).

[1] Rusinov, A. I. Superconductivity near a Paramagnetic Impurity. *JETP Lett.* **9**, 85 (1969).

[2] Ruby, M., Heinrich, B. W., Peng, Y., von Oppen, F. & Franke, K. J. Wave-Function Hybridization in Yu-Shiba-Rusinov Dimers. *Phys. Rev. Lett.* **120**, 156803 (2018).

[3] von Oppen, F. & Franke, K. J. Yu-Shiba-Rusinov states in real metals. *Phys. Rev. B* **103**, 205424 (2021).

[4] Steiner, J. F., Mora, C., Franke, K. J. & von Oppen, F. Quantum magnetism and topological superconductivity in Yu-Shiba-Rusinov chains (2021). 2107.00031.

[5] Liebhaber, E. *et al.* Yu–Shiba–Rusinov States in the Charge-Density Modulated Superconductor $NbSe_2$. *Nano Lett.* **20**, 339–344 (2020).
